# Analysis tools for single-monomer measurements of self-assembly processes

**DOI:** 10.1038/s41598-022-08245-6

**Published:** 2022-03-18

**Authors:** Maria Hoyer, Alvaro H. Crevenna, Radoslaw Kitel, Kherim Willems, Miroslawa Czub, Grzegorz Dubin, Pol Van Dorpe, Tad A. Holak, Don C. Lamb

**Affiliations:** 1 Department of Chemistry, Center for NanoScience, Nanosystems Initiative Munich (NIM) and Center for Integrated Protein Science Munich (CiPSM), Ludwig-Maximilians University Munich, Munich, Germany; 2 Epigenetics and Neurobiology Unit, EMBL Rome, Monterotondo, Italy; 3 Department of Organic Chemistry, Faculty of Chemistry, Jagiellonian University, Gronostajowa 2, 30-387 Krakow, Poland; 4 Malopolska Centre of Biotechnology, Jagiellonian University, Gronostajowa 7a, 30-387 Krakow, Poland; 5 imec, Kapeldreef 75, 3001 Leuven, Belgium

**Keywords:** Biophysics, Computational biology and bioinformatics, Molecular biology, Nanoscience and technology

## Abstract

Protein assembly plays an important role throughout all phyla of life, both physiologically and pathologically. In particular, aggregation and polymerization of proteins are key-strategies that regulate cellular function. In recent years, methods to experimentally study the assembly process on a single-molecule level have been developed. This progress concomitantly has triggered the question of how to analyze this type of single-filament data adequately and what experimental conditions are necessary to allow a meaningful interpretation of the analysis. Here, we developed two analysis methods for single-filament data: the visitation analysis and the average-rate analysis. We benchmarked and compared both approaches with the classic dwell-time-analysis frequently used to study microscopic association and dissociation rates. In particular, we tested the limitations of each analysis method along the lines of the signal-to-noise ratio, the sampling rate, and the labeling efficiency and bleaching rate of the fluorescent dyes used in single-molecule fluorescence experiments. Finally, we applied our newly developed methods to study the monomer assembly of actin at the single-molecule-level in the presence of the class II nucleator Cappuccino and the WH2 repeats of Spire. For Cappuccino, our data indicated fast elongation circumventing a nucleation phase whereas, for Spire, we found that the four WH2 motifs are not sufficient to promote de novo nucleation of actin.

## Introduction

**Figure 1:**
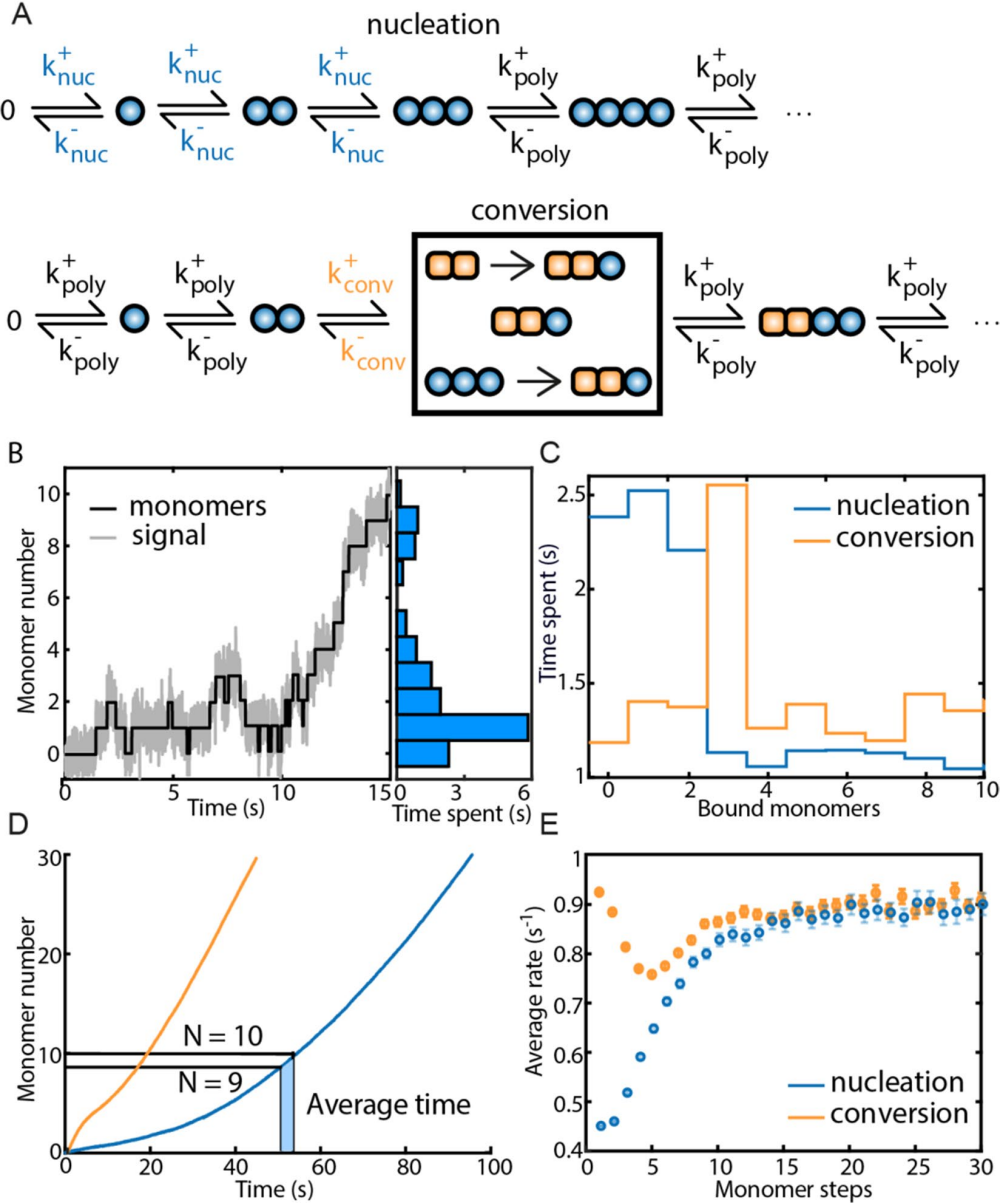
Analysis methods for single-filament data of protein self-assembly. (**A**) Kinetic model of the simulated nucleation and conversion process. For nucleation, k^+^_nuc_ and k^−^_nuc_ are changed to k^+^_poly_ and k^−^_poly_ as soon as the nucleus size is reached (here three monomers). For a conversion mechanism, only the assembly step associated with conversion (i.e., for example a restructuring of the assembled oligomer) shows k^+^_conv_ and k^−^_conv_. Earlier and later steps follow k^+^_poly_ and k^−^_poly_. For the conversion step, three main possibilities could lead to the slower kinetics as indicated by the black box: (1) the pre-existing oligomer needs to rearrange prior to the next binding event, (2) the restructuring occurs upon binding or (3), the restructuring occurs after binding of the next monomer. All possibilities result in a slower kinetic step and are treated the same in the simulations. (**B**) Example trace of a simulated growth process undergoing a nucleation mechanism with a nucleus size of four monomers (black) with Gaussian noise (grey). The distribution of time spent at a certain oligomer size (blue histogram) contains information about the nucleation mechanism when it is applied to many single oligomer traces (**C**). (**C**) Visitation analysis on a nucleation (blue) or a conversion mechanism (yellow). The visitation analysis identifies the nucleation mechanism as well as the nucleus size for nucleation or conversion. (**D**, **E**) Average rates analysis. (**D**) Average traces of a nucleation (blue) or conversion (yellow) mechanism. From the average trace of many individual filaments growing from the same starting point, average rates can be calculated from the average time it takes to reach the next oligomer size. (**E**) Average rates plotted versus the monomer number (oligomer size). Different nucleation mechanisms show individual signatures. The nucleation and conversion kinetics were simulated to show slower association rates than the polymerization kinetics.

Protein polymerization plays an important role in both pathological and physiological processes. For example, polymerizing proteins, such as actin, build up the cytoskeleton and are of vital importance for the cell. At the same time, the aggregation of misfolded proteins is associated with Alzheimer’s disease or type II diabetes mellitus^1^. Despite the recent advances in our understanding of elongation processes, many of the details during the early nucleation phases of polymerization are still unknown. As a growing number of proteins are shown to polymerize^2,3^, tools to elucidate the mechanism of nucleation become more relevant. In general, nucleation mechanisms are divided into primary and secondary nucleation processes. Primary nucleation is initiated by isolated monomers whereas secondary nucleation requires an initial filament. Most primary nucleation processes studied so far^4^ have been classified to occur either through a nucleation-elongation mechanism or through a conversion mechanism (Fig. 1A). For example, actin polymerization has served as a canonical representative of nucleation-elongation^5^, although this may depend on the polymerization conditions^6^, whereas amyloid formation has been reported to follow conversion models^7–9^. Therefore, analysis tools that elucidate the nucleation mechanism are of wide interest.

Of the various methods available to investigate nucleation, single-molecule techniques are a promising route since they can provide direct, real-time measurements of single monomer association and dissociation events occurring during the growth process^10^. In particular, single-molecule fluorescence techniques combined with the use of zero-mode waveguides (ZMW)^11^ have opened the possibility to observe the early stages in oligomer assembly directly. Similarly, single events such as binding and dissociation of monomers on individual oligomers can be measured via scattering^12^. By using single-molecule techniques, binding events can be visualized as step-wise signal increases over time and dissociation events show a step-wise decrease in signal (Fig. 1B). In an often used approach, the residence-time at each monomer number is pooled together and typically than fit to an exponential function to determine the lifetime of the oligomer at a particular monomer number. This is repeated for all monomer numbers. This method is often referred to as a dwell-time-analysis and has been applied in many biophysical studies^13,14^. The dwell-time-analysis provides the microscopic rate for each step and can, therefore, reveal the unique underlying mechanism. However, the accuracy of the analysis, and thereby the identification of the nucleation mechanism, depends on the quality of the data and sufficient sampling of the different steps. For example, to estimate the rate of a single transition accurately, a sampling rate has to be chosen that is at least ten times as fast as the rate to be estimated. For some techniques such as optical tweezers, the sampling frequency is not a problem as it is only dictated by the readout speed of a position-sensitive detector. However, getting sufficient statistics is more difficult due to the low throughput of these types of measurements. For fluorescent samples, high sampling frequency comes at the expense of increased noise and/or an increased probability of photobleaching, which complicates the analysis and shortens the total experimental time.

Besides the wealth of data provided by single-molecule techniques, information on the underlying mechanism of nucleation and growth can be extracted equally from the average time-course of filaments growing on many surface-bound nucleator proteins, for example, by using total-internal reflection microscopy (TIRFM)^15^, circular dichroism^16^, or other spectroscopy methods^17–19^. By only measuring filaments growing via the nucleator protein of interest tethered to the surface, spontaneous oligomerization events occurring in solution can be excluded. This approach provides the possibility to synchronize the starting point of the oligomerization process.

In this work, we developed and tested analysis methods for kinetic data of filament growth on the single oligomer level as well as for averaged but synchronized filament growth. Our objective is to (1) recover the correct on- and off-rates of the single monomer addition and dissociation events, and (2) recover the correct assembly mechanism via the relative differences between monomer binding events at different oligomer sizes. Thereby, we wish to distinguish between the two main mechanisms: a nucleation mechanism with a slow on-rate or high off-rate in the beginning until the polymerization phase is reached, and a conversion mechanism where a single slow on-rate or high off-rate defines a bottleneck for successful polymerization (Fig. 1A). We tested our developed methods by simulating the growth processes of single oligomers with defined binding and dissociation rates of the monomers, dependent on the oligomer size at the time of the binding or dissociation event (see “Materials and methods” and Supplementary Table S1).

To test the robustness of the analysis tools, we investigated the influence of the SNR, the simulated measurement rate and the relative difference between the nucleation and polymerization kinetics. Furthermore, since measurements in ZMW and TIRFM depend on dye-labeled monomers, we looked into the distribution of labels per monomer (i.e., the labeling efficiency), as well as the photobleaching of fluorescent labels. Finally, we applied the analysis tools on experimental data of actin nucleation using the formin-homology domain 2 (FH2) of Cappuccino and the WH2 domains of Spire (Spire-ABCD) as nucleators and compared the results to simulations of unhindered and unsuccessful oligomer growth. For Cappuccino, the data indicated a circumvention of the nucleation phase resulting in unhindered growth as expected. For Spire-ABCD, we found that the WH2 domains are not sufficient to promote actin nucleation starting from the purely monomeric species.

## Theory and simulations

For the development and testing of different analysis methods, we simulated the assembly of single monomers into filaments by sequential monomer addition based on the basic principles of filament formation^20^. The assembly process is ruled by the kinetics of individual binding and dissociation events. We looked into the two major mechanistic possibilities that have been found to occur during primary nucleation^21^. First, we looked into a one-step nucleation mechanism, i.e., the formation of a nucleus of defined size (n monomers) that represents the smallest stable structure and allows subsequent polymerization. Hereby, every monomer binding event until the formation of the nucleus is defined by the nucleation kinetics, and the faster polymerization kinetics take effect after the nucleus size has been reached (Fig. 1A). To simulate slower nucleation kinetics, either the dissociation rate constants of the single monomers can be enhanced, or the association rate constants can be slowed down. It is typically assumed that the association rate constants do not change during a nucleation process^22^. However, we also looked into the effect of association rates as well to include all possibilities. The second possible mechanism that we investigated is that of a conversion step. Here, a small oligomer of a particular size undergoes a conformational rearrangement that typically leads to a decrease in the dissociation rate. Hence, once this conformational change, i.e. conversion step, has taken place, polymerization is observed. We tested the different analysis methods for their ability to^1^ detect a transition from nucleation-governed kinetics to faster polymerization and (2) to detect a single slow conversion step and thereby distinguish between the two mechanisms.

### Simulations

We performed stochastic simulations of an assembly process with individual on- and off-rates for each monomer step (Fig. 1A, Supplementary Table S1). The time spent at the current oligomer size as well as whether the next step was an association or dissociation event was randomly selected from an exponential distribution based on the on- or off-rates for the respective oligomer size. This process was continued for each oligomer until the total preselected simulation time was exceeded. Based on the association and dissociation events, a monomer number versus time trace was built using a sampling rate of 100/s, unless stated otherwise. The selected sampling rate roughly corresponds to the measurement rates of ~ 10 ms/frame currently achievable with modern cameras. For the nucleation mechanism, the kinetics before reaching the nucleus size were defined by the monomer association rate k^+^_nuc_ and the dissociation rate k^−^_nuc_, which were treated as identical for oligomers smaller than the nucleus size. When the monomer number reached the nucleus size, the kinetics changed to the polymerization rates k^+^_poly_ and k^−^_poly_. The second mechanism, a conversion step, was simulated using a single association rate (k^+^_poly_) and a single dissociation rate (k^−^_poly_) for all monomers with the exception of the conversion step, which shows a slower kinetics k^+^_conv_ and k^−^_conv_. If not stated otherwise, k^+^_poly_ was set to 1 s^−1^ and k^−^_poly_ to 0.1 s^−1^, which corresponds to a factor of 100 fold and 1000 fold the sampling time, respectively (Supplementary Table S1). These rates are in the range of the known rates of pointed end actin polymerization^23^. Looking at the relative difference between the expected rates and the sampling rate, our results can be used to estimate the necessary sampling rate for an experiment of this kind. In addition, the results can be further evaluated with respect to the sampling rate to determine whether the extracted rates are trustworthy. For k^+^_nuc_ and k^+^_conv_, we chose values that corresponded to 10%, 50%, or 80% of k^+^_poly_ to test the sensitivity of the analysis methods for detecting slight changes in the kinetics. Accordingly, we chose k^−^_nuc_ and k^−^_conv_ to be 2-, 5-, or 10-fold faster than k^−^_poly_. An overview of the chosen simulation parameters can be found in Supplementary Table S1.

The results of the simulated assembly process were transformed into fluorescence traces by overlaying them with Gaussian noise using a signal-to-noise ratio (SNR) of 2, unless stated otherwise (Fig. 1B). We analyzed both single filament traces as well as an average of 1000 traces from individual simulations (Fig. 1D). For the single filament traces, we extracted the underlying monomer versus time trace via a step-finding algorithm (see “Materials and methods” for details)^6^. For slow sampling rates, two fast consecutive association steps could appear as one step with a double step size. Therefore, we used the mode of the step sizes to determine the average intensity of a monomer,  identified double and triple steps, and corrected the monomer number accordingly.

### Visitation analysis and average rates

As an alternative to the dwell-time analysis, we present two novel analysis tools for investigating the microscopic mechanism underlying filament formation: the visitation analysis and the average-rate analysis. The visitation analysis samples the time the oligomers spend at each oligomer size (Fig. 1B,C). The resulting histogram contains information regarding the different rates involved in the single steps of the nucleation process. Steps that are faster or slower than that of their neighboring oligomers are directly visible (Fig. 1C). For a polymer that forms via a nucleation-elongation mechanism, the most often detected oligomer sizes are determined by the nucleation phase where oligomers fluctuate between sizes equal to or below the nucleation size. The initial oligomers form and immediately disassemble due to the high thermodynamic barriers that need to be overcome. Upon successful formation of the nucleus, the kinetics change and elongation occurs. As k^+^_poly_ is typically ≫ k^−^_poly_, the oligomers do not spend much time at specific monomer sizes just above the nucleation size as k^+^_poly_ dominates and the oligomers grow (Fig. 1C blue line). For a filament that polymerizes through a conversion mechanism, a slow conversion step at a particular monomer number size is required for elongation. Hence, the oligomers spend a significant fraction of the time at this monomer size in comparison to the fast fluctuations at smaller oligomer sizes and the mostly continuous elongation observed upon undergoing the conversion step (Fig. 1C orange line).

The second approach is the average-rate analysis. Here, the rates for each monomer addition are calculated from the time it takes for the average trace of hundreds to thousands of growing oligomers to reach the next monomer number (Fig. 1D). These average rates are then plotted as a function of oligomer size (Fig. 1E). For many oligomers that started growing simultaneously, a nucleation mechanism is directly visible in the shape of the averaged intensity curve since the slope of the curve is less steep in the beginning of the assembly process due to the slower nucleation kinetics (Fig. 1D). Since the sum follows the same behavior as the average of many traces, this approach can also be applied to experiments that are synchronized but where single-filament data is not available. Averaging is a useful tool to circumvent the limitations of a low SNR. When single filament data is available, the average intensity from many single-filament traces can still be calculated and both analyses can be performed. Experimentally, the average signal for the addition of a monomer needs to be known to convert the intensity information into a monomer number. For a growth process that does not reach high monomer numbers on average, fractional average rates in 0.1 monomer steps can also be extracted.

Similar to the visitation analysis (Fig. 1C), the average rate (Fig. 1E) also reveals signatures that can be associated with either a nucleation-elongation (Fig. 1E blue points) or a conversion mechanism (Fig. 1E orange points). Nucleation-elongation starts from a low average rate and monotonically increases until it reaches the average elongation rate reflecting the transition from the slow nucleation phase to the faster elongation regime. The average rate from a conversion mechanism first drops around the oligomer size where the conversion occurs and then increases until it also reaches the average elongation rate.

As a frequently employed standard analysis tool, we also tested the dwell-time analysis for its functionality on single-oligomer data. The dwell-time analysis generates a histogram of waiting times until the next monomer binding event occurs for oligomers of different sizes (Fig. S1A,B). An exponential fit of the distribution yields the rate of the respective monomer binding event. The on- and off-rates were extracted from an exponential fit of the dwell-time distribution (Fig. S1) and plotted against the monomer number to visualize the transition from nucleation to polymerization that occurs once the nucleus size is reached. This approach is very powerful and, when the experimental data allows extraction of the correct rates, the nucleation mechanism can be elucidated.

### Distinguishing between nucleation and conversion mechanisms

**Figure 2:**
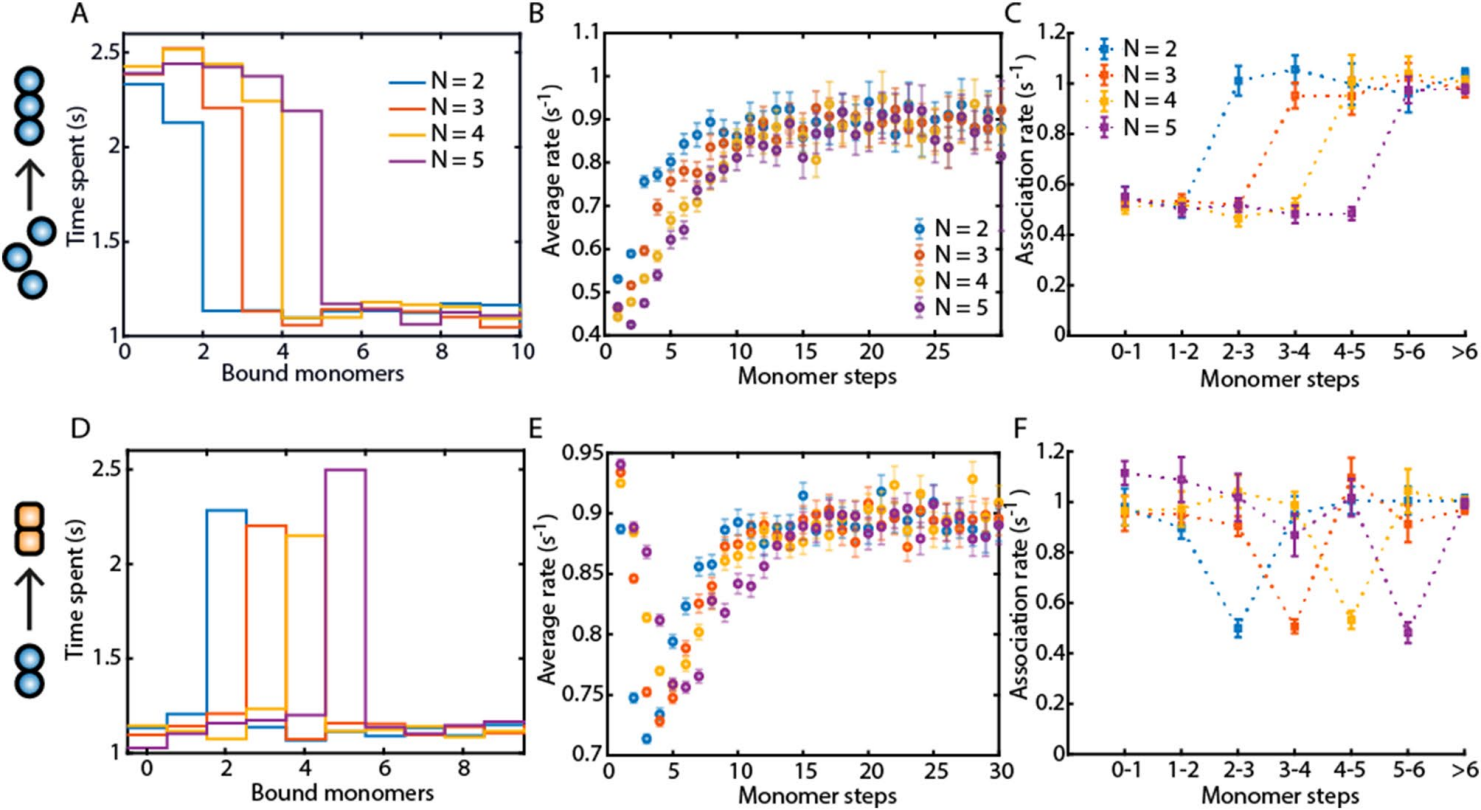
A comparison of the visitation analysis, average rates and dwell-time analysis on an assembly process with nucleus or conversion step with a size of 2, 3, 4 or 5 monomers. (**A**, **D**): A visitation analysis on a nucleation (**A**) and on a conversion mechanism (**D**). (**B**, **E**): Average rate analysis on the same nucleation (**B**) and conversion mechanism (**E**) as in (**A**, **D**) . (**C**, **F**): Dwell-time analysis on the same nucleation and conversion mechanism as in (**A**, **D**). The dwell-time analysis can identify a mechanism with a change in the on-rate by extracting the association rates (**C, F**), but not a mechanism with a change in the off-rate, since the extracted dissociation rates do not indicate any changes in the simulated rates (see Fig. S2). Error bars for the rates represent the 95% confidence intervals of the exponential fits. The association rates during nucleation k^+^_nuc_ or the conversion step k^+^_conv_ were chosen to be 50% of k^+^_poly_ with no change in the off-rates.

To determine how well the different analysis approaches can distinguish the mechanism of nucleation, data were simulated for both a nucleation mechanism and a conversion mechanism occurring at an oligomer size of 2, 3, 4 and 5 monomers. The visitation analysis and the average-rate analysis are both able to identify the nucleus size and a conversion step accurately, independent of whether the association or the dissociation rate is changed (Fig. 2, Fig. S2). The dwell-time analysis can identify a change in the association rate for both the nucleation and conversion mechanism (Fig. 2C,F, Fig. S1). For both nucleation and conversion, the dwell-time analysis is not sensitive towards a change in the dissociation rates (Figs. S1D,F, S2C,F).

During a nucleation process or around a conversion step, the self-assembling oligomers are likely to fluctuate around the rate-limiting steps until the nucleus size is reached or the necessary rearrangement has occurred. To quantify these dynamics, we calculated the number of upward and downward transitions that start from a certain monomer number (additional information provided by the visitation analysis). With this approach, a nucleation mechanism can be visualized (Fig. S3A,B). Before reaching the nucleus size, multiple fluctuations upwards and downwards are observed whereas, after nucleation, only elongation (upward steps) is observed. However, the difference in the transitions around one single step is not sufficient for the detection of a conversion step with this approach (Fig. S3C). Furthermore, the number of visits or the meantime per visit were calculated as an alternative to calculating the mean time the oligomers spent at a certain oligomer size. Both approaches could identify a change in the on- and off-rates of individual steps similar to the visitation analysis (Fig. S4).

Since the visitation analysis could detect conversion steps with high sensitivity, we wanted to further test its ability to identify single steps with different kinetic rates. Therefore, we simulated a growth process with individual slower or faster steps at defined oligomer sizes as depicted in Fig. S4A. Here, the visitation analysis is sensitive to changes in the on and off-rates of individual steps (Fig. S4B), even with only slight differences in the rates (Fig. S5A). The dwell-time analysis could not identify even one of the faster or slower kinetic steps (Fig. S4E). Thus, the visitation analysis could identify even multiple conversion steps during an assembly process.

A nucleation phase is characterized by a slower on-rate respective to the polymerization kinetics, or by a destabilization effect via high off-rates. These two mechanisms are visible in the average rates (Fig. S6). A slower on-rate during nucleation results in a steady increase in the average rates with oligomer size (Fig. S6A) whereas, with a faster off-rate, the average rates first decrease with oligomer size and then increase again (Fig. S6B). Therefore, the average rates can be used to determine whether the association or the dissociation kinetics are changed when the nucleus size or conversion step is reached.

### Influence of SNR and data collection rate

Every analysis tool has its requirements and limitations depending on the experimental data. The requirements are coupled to the question that one wishes to address: do we want to determine the nucleation mechanism or is the aim to correctly determine every microscopic rate for each step? To estimate the required SNR and measurement rate for correctly answering these questions, we tested the presented analysis methods for their robustness as a function of SNR and sampling times. The average-rate analysis proved to be the most robust towards low SNR and slow data collection rates (Fig. S7). The visitation analysis (Figs. S5, S8) and the dwell-time analysis (Fig. S9) both use single-filament data and a step-finding algorithm. However, the visitation analysis is much more robust towards SNR and slow measurement times. In addition, it is able to detect multiple individual steps even at a SNR of 0.5 and a measurement rate of 5 times the fastest on-rate (Fig. S5). The visitation analysis is also very robust towards lower SNR and slower measurement rates when detecting a nucleation mechanism (Fig. S8, Table 1).

**Table 1:** Robustness of the analysis methods.

	Dwell-time analysis		Visitation analysis		Average-rate analysis	
	Recommended conditions for distingushing					
	The mechanism	Single steps	The mechanism	Single steps	The mechanism	Single steps
SNR	> 1	> 1	> 0.1	> 1	> 0.1	> 0.1
Sampling rate	> 10×^a^	> 20×	> 1×	> 2×	> 1×	> 5×
LE (stochastic)	> 0.7	> 1	< 1	1	> 0.3	> 0.7 (1^b^)
LE (specific)	> 0.3	1	> 0.3	1	> 0.3	1
Photobleaching	< 3%^a^	< 0.1%	3%	< 2%	< 3%	< 2%

^a^Compared to the fastest rate.

^b^1 % to obtain the correct conversion site.

Using a multi-step correction, we could improve the performance of the dwell-time analysis also for measurements with a slow data collection rate (Fig. S9H). The approach uses the intensity of the steps to identify single, double or triple steps (see “Materials and methods” for details) and to correctly transform this information into the appropriate number of monomers, even when the individual single steps cannot be resolved.

### Effect of labeling efficiency and photobleaching

Imaging of filament growth in ZMW or TIRFM relies on fluorescent labeling of the monomers. Labeling of the proteins for the nucleation studies brings additional complexities into the analysis. Dependent on whether a stochastic or a specific labeling strategy is used, the fraction of labeled monomers can either show a Poisson distribution of labels, where there may be more than one dye per monomer, or each monomer has either zero or one dye molecule attached as only one binding site is present when specific labeling approaches are used. Though high-labeling efficiencies are, in some cases, possible e.g.^24^, sample preparation can be very time-intensive. At times, it is also necessary to use a mixture of labeled and unlabeled monomers to avoid influencing the assembly process. When the protein is not specifically labeled with 100% labeling efficiency, the labeling efficiency needs to be accounted for.

Proteins are typically labeled stochastically on naturally occurring lysine or cysteine residues, which results in a Poisson distribution of labels. In this case, one monomer may contain more than one dye molecule. When only one labeling site is available, the monomers can be specifically labeled resulting in zero or one fluorophore per monomer. Hence, after simulating a monomer trace for the given conditions, we modified the trace assuming either a Poisson distribution of different labeling efficiencies from 0.3 to 3, or with a specific labeling approach with labeling efficiencies from 0.3 to 1 (Fig. S10). For determining the nucleation mechanism, labeling efficiencies below 100% do not provide any difficulties for all three analysis methods (Fig. S10A–F). For detecting single steps, however, the dwell-time analysis and visitation analysis require high labeling efficiencies (Fig. S10G–M). Only the average-rate analysis can deal with labeling efficiencies down to 30% (Fig. S10H,L). For the dwell-time and the average-rate analysis, the extracted rates for the polymerization regime corresponding to k^+^_poly_ can be corrected using LE* k^+^_poly_ as the extracted polymerization rates stagnate at this level (Fig. S10). Thus, if the process reaches polymerization kinetics and the labeling efficiency is known, the correct polymerization rate can be extracted quantitatively.

Another artifact that impacts the measurements is photobleaching of the fluorophores. Although photobleaching can be reduced using oxygen scavenging systems, it cannot be completely avoided and will have an impact on the intensity signal depending on the photobleaching rate. In intensity traces, a photobleaching step is indistinguishable from a dissociation step. This hinders not only the direct measurement of the dissociation rate in single-filament traces but also leads to a mismatch between the intensity level and the monomer number, which influences the extracted on-rates as well. To test for the influence of photobleaching, we introduced down steps in the simulations based on different photobleaching rates. These rates are in the regime of experimentally determined values (^6^, Fig. S11). When a monomer dissociates after photobleaching, no down step is introduced. If not indicated otherwise, photobleaching was applied to 100% specifically labeled monomers.

With respect to distinguishing between different mechanisms of nucleation, the presented analysis methods are all affected by photobleaching, though some still correctly identify the underlying mechanism (Table 1). For all analysis methods, photobleaching should not exceed 3% of the on-rate during polymerization or 30% of the off-rate (Figs. S7F, S8G,H, S12). For the average-rate analysis, the apparent average rates can decrease with oligomer size due to the effect of photobleaching. However, the signature of a nucleation or conversion mechanism is still visible at a photobleaching rate of 3% of the simulated on-rate (Fig. S7G,H). The visitation analysis of a growth process that assembles fast compared to photobleaching is largely unaffected (Fig. S8F).

Using the dwell-time analysis, the influence of photobleaching can also be used as a tool by comparing measurements with different photobleaching rates. In this way, it is possible to estimate the nucleation or conversion step despite photobleaching (Fig. S12). A high off-rate leads to the exchange of photobleached monomers with unbleached monomers. By comparing the apparent off-rates of measurements with different photobleaching rates as determined by the dwell-time analysis (Fig. S12B,E), the nucleus size or a conversion step could be correctly determined even at higher photobleaching rates (Fig. S12C,F).

**Figure 3:**
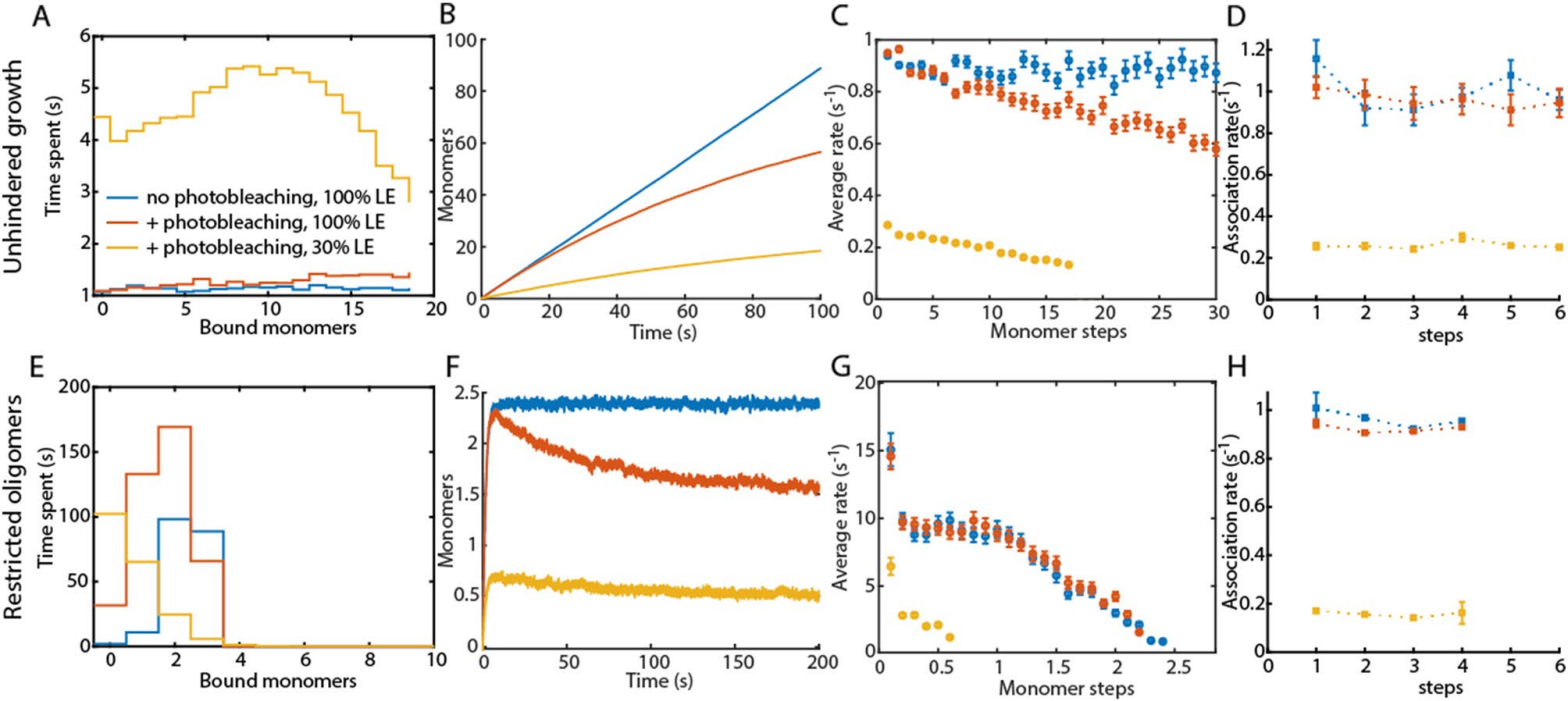
Effect of photobleaching and labeling efficiency on unhindered growth and restricted growth that stops after the assembly of four monomers. Both unhindered (**A**–**D**) and restricted growth (**E**–**H**) were simulated using k^+^_poly_ = 1/s and k^−^_poly_ = 0.1/s. The visitation analysis (**A**, **E**), average rates (**C**, **G**) extracted from the average traces (**B**, **F**) and dwell-time analysis (**D**, **H**) were applied to data with 100% labeling efficiency and no photobleaching (blue data). The average trace of restricted growth reaches only 2.5 monomers, despite possible growth until 4 monomers, because of the equilibrium between the on- and the off-rate. The effect of a photobleaching rate of 0.01/s does not significantly influence the extracted rates and the visitation analysis (red data). The combination of stochastic labeling of 30% and photobleaching with a rate of 0.01/s affects all analysis methods (yellow). Error bars for the dwell-time analysis represent the 95% confidence intervals of the exponential fit.

To visualize the combined effect of labeling efficiency and photobleaching on different oligomer growth scenarios, we simulated unhindered filament growth without nucleation or conversion and filaments whose growth was restricted to a tetramer (Fig. 3). A photobleaching rate at 10% of the polymerization kinetics still allows correct kinetic analysis via the visitation analysis, dwell-times analysis and average rates (Fig. 3 red curves). In combination with a stochastic labeling efficiency of 30%, the visitation analysis shows a distribution shifted towards smaller oligomers sizes (Fig. 3A,E). The dwell-time analysis resulted in reduced rates (Fig. 3B,F). For a restricted growth mechanism where the growth stops upon obtaining a certain oligomer size (exemplified for a tetramer in Fig. 3E–H), the average rates can only be extracted for the very first monomers because the average filament size does not reach beyond 0.5 monomers (Fig. 3G). For that reason, fractional average rates in 0.1 monomer steps have been extracted (Fig. 3H).

### Experimental results

We used the three analysis methods (the dwell-time analysis, visitation analysis, and fractional average-rate analysis) to investigate the nucleation mechanism of the actin nucleators Cappuccino and the ABCD fragment of Spire. The formin Cappuccino stabilizes actin monomers via its FH2 domains, thereby promoting nucleation^25–27^. Spire-ABCD contains 4 Wiskott–Aldrich syndrome protein (WASP) homology 2 (WH2) domains, labeled as ABCD, which bind actin monomers^28,29^. For the experiments, 30% stochastically labeled G-actin-Cy5 was used. The biotinylated nucleator proteins were attached to the bottom of zero-mode waveguides via a biotin-streptavidin interaction, and the actin monomers were added directly after the start of the measurement (see “Materials and methods” and Ref.^6^ for details). Actin-Cy5, as well as biotinylated Cappuccino and ABCD1 domain of Spire, were fully functional (Figs. S13, S14).

**Figure 4:**
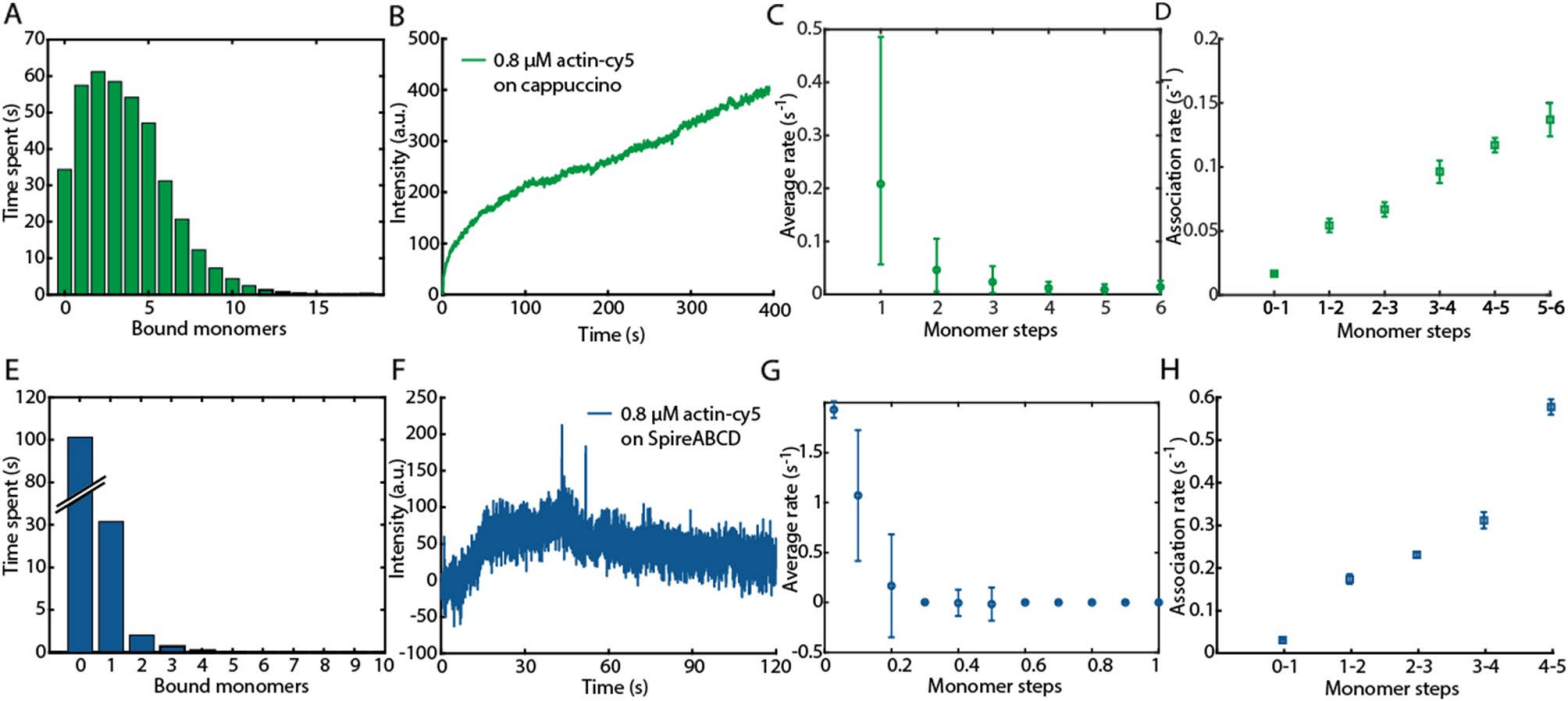
Experimental data of 30% stochastically labeled actin-Cy5 growing on Cappuccino and Spire-ABCD. A comparison between a visitation analysis, dwell-time analysis and average rate analysis is performed for the measurements with Cappuccino (**A**–**D**, green) and Spire-ABCD (**E**–**G**, blue). (**A**, **E**): The visitation analysis, (**B**, **F**) average intensity, (**C**, **G**) fractional average-rate analysis and (**D**, **H**) dwell-time analysis showing the first 5 monomer association steps from 700 individual traces are plotted. The fractional average rates have been calculated using the time until the average trace reached the intensity change corresponding to 0.1 monomers (i.e. between 0 and 0.1, between 0.1 and 0.2, between 0.2 and 0.3, etc.). Fractional average rates are 10 times faster than average rates because the average intensity of only 1/10 of a monomer has to be reached. The experimentally determined photobleaching rate was 0.025/s.

For actin growth on the strong nucleator Cappuccino (Fig. 4A–D), we observed an increase in the average filament size over time (Fig. 4B) reaching oligomer sizes of up to 10 monomers already in the first 400 s (Fig. 4A). The dwell-time analysis showed increasing rates with each step (Fig. 4D). The fractional average rate analysis, however, shows a decrease in the association rates with oligomer size. This observation can be explained by the influence of a labeling efficiency of 30% and photobleaching. Thus, even a simulation of unhindered growth, i.e. no nucleation or conversion mechanism, under these conditions showed decreasing average rates with oligomer size (Fig. 3C).

Measurements of actin nucleation in the presence of Spire-ABCD (Fig. 4E–H) did not show prominent growth in the average filament trace (Fig. 4F), even though the dwell-time analysis showed increasing on-rates with oligomer size (Fig. 4H). In contrast, the visitation analysis showed that only very few monomers bind to Spire-ABCD (Fig. 4E). The discrepancy between the dwell-time analysis and the other two approaches, i.e. the visitation analysis and average-rate analysis, can be explained by a bias in the on-rates. When filament growth is very unlikely, only fast on-rates can lead to the assembly of higher oligomers, thus filtering the distribution of individual on-rates for the fast rates. This affects the dwell-time analysis, but not the visitation and average-rate analysis. Comparing the resulting actin growth on Spire-ABCD (Fig. 4E-H) with simulated data affected by photobleaching and labeling efficiency (Fig. 3E-H), a restricted growth model up to a few monomers could reasonably describe the data.

## Discussion

Single filament data contain information regarding the nucleation mechanism that is not immediately visible from inspection of the recorded time traces. The most common approach for dealing with data showing the individual association and dissociation steps is the kinetic analysis of the dwell-times. The obtained microscopic rates should directly reflect the underlying self-assembly mechanism. However, the correct interpretation of dwell-time distributions depends, to a high extend, on the quality of the data (i.e. signal-to-noise ratio, SNR) and also on the measurement rate or the influence of dye photobleaching in fluorescence microscopy measurements (Table 1). Therefore, we developed more robust analysis methods that give insights into the microscopic mechanism of the self-assembly process that are less prone to misinterpretation. An overview of the minimal requirements for each of the tested analysis methods can be found in Table 1—with respect to the measurement rate, SNR, labeling efficiency and photobleaching rate.

The dwell-time analysis provides the microscopic rates for the addition of each monomer and should, therefore, reveal the underlying assembly mechanism. Under the appropriate conditions, this method works well for the extraction of association rates (Fig. S1C,E). However, it is very sensitive to the SNR and acquisition speed, which needs to be more than an order of magnitude faster than the expected kinetics (Table 1). Also, photobleaching leads very quickly to a loss of synchronization between the measured and actual filament size. However, one can also apply photobleaching as a tool by measuring filament formation with different photobleaching rates. A comparison of the extracted rates can help to identify the nucleus size or conversion step (Fig. S12).

The visitation analysis is able to detect a slow nucleation phase despite non-ideal measurement conditions like low SNR (Fig. S8). Moreover, it is very suitable to identify multiple events with slower or faster kinetics during an assembly process (Fig. S5). For the visitation analysis, the sampling rate still needs to be about a factor of 2 higher than that of the expected kinetics. However, this is an order of magnitude slower than the required speed for the dwell-time analysis (Table 1). Because of the direct read-out of the monomer number, the visitation analysis works best with high labeling efficiencies, especially for the detection of a single conversion step (Table 1). However, it can detect a nucleation or conversion mechanism also for lower labeling efficiencies and is less sensitive to stochastic labeling compared to the dwell-time analysis. The visitation analysis on single-filament data is, therefore, a suitable method for detecting nucleation or individual conversion steps, especially at low SNR or slower sampling speeds (Table 1).

When the quality of single-filament data is not sufficient for the use of a step-finding algorithm to extract the individual monomer binding and dissociation events, an average trace can be obtained from the growth information of multiple filaments. When the number of filaments and the intensity information of a single monomer binding event is known, average rates can be used to visualize and detect a slow nucleation phase or a pronounced slow conversion step. The average-rate analysis can deal with a sampling time in the same range as the expected kinetics and a SNR as low as 0.1 (Fig. S7, Table 1). For the detection of a nucleation or conversion mechanism, also the labeling efficiency can go down to 30%. However, when the correct oligomer size for the nucleation or conversion event needs to be elucidated, a labeling efficiency of 100% is necessary. The average rate analysis is the most robust analysis method present here.

For the simulations of a nucleation or conversion process, changes in the dissociation and the association rates have been separated to study the effects independently. When only the association rate or the dissociation rate is affected during the growth process, the average rate analysis can be used to determine which rate changes when the nucleus size is reached (Fig. S6).

Photobleaching, in general, affects all analysis methods (Table 1). The dwell-time analysis is most affected by photobleaching as the kinetics of single steps has to be determined (Fig. S9, Table 1). The visitation analysis and the average-rate analysis require a photobleaching rate that should not exceed 2–3% of the association rate (Table 1). The labeling efficiency should be at least 30–50%, depending on the analysis method used and whether stochastic or specific labeling is used (Fig. S10, Table 1). The visitation analysis allowed for labeling efficiencies down to 30% for the detection of a nucleation mechanism. In contrast, the dwell-time analysis reproduced the correct on-rate only with very high labeling efficiencies. In the case of specific labeling of the monomers, a nucleation process could still be identified even at 30% labeling efficiency with all three presented methods. A specific conversion-step could only be detected with the correct oligomer size for a specific labeling efficiency of 100%. Hereby, the influence of incomplete labeling on the analysis results increases with higher oligomer numbers since the potential discrepancy between the true monomer number and the intensity-based monomer number increases with the oligomer size. However, the existence of a conversion step, without a clear indication of the oligomer size, could be detected at lower labeling efficiencies (Fig. S10).

When analyzing experimental data, such as the actin growth on Spire-ABCD and Cappuccino shown here, the experimental details like sampling rate, labeling efficiency and photobleaching have to be taken into account. To check the influence of the sampling rate on the dwell-time analysis experimentally—as carried out in the simulations (Fig. S9)—we increased the sampling rate for the experiments with Cappuccino from 5 to 16.6 Hz. This led to an increase of the estimated association rate (0.24/s or 0.72/s when accounting for the labeling efficiency, Fig. S15C). However, the extracted rates are still slower than the expected 8/s for 800 nM actin^23,30,31^. For association rates on the order of 10/µM/s, a monomer concentration of 0.8 µM and a labeling efficiency of 30%, the minimal sampling rate for the dwell-time analysis is 48/s (20*0.3*8/s = 48/s), as the dwell-time analysis yields trustworthy rates at > 20 times the expected association rate (Fig. S9). These results suggest that the current time resolution used in these experiments is insufficient for an accurate estimation of the association rates via a dwell-time analysis. The visitation analysis and the average trace, however, clearly identify fast actin assembly as expected (Fig. 4).

The photobleaching rate of actin-Cy5 showed a double exponential decay as expected for Cy5^32^ (Fig. S11). The slower rate was at 0.025/s, which corresponds to 3.5% of the extracted on-rate corrected for the labeling efficiency (0.72/s). That corresponds to the highest photobleaching rate used here in simulations (3% of the on-rate). For the average-rate analysis, a photobleaching rate of 3% results in a decrease and even a cut-off of the extracted rates even for unhindered growth (Fig. 3). This explains the decrease of the average rates for actin-Cy5 growing on Cappuccino (Fig. 4C). Thus, when considering the expected effects of the labeling efficiency and photobleaching on the experimental data, the dwell-time analysis, visitation analysis, and average-rate analysis indicate continuous growth for actin growing on Cappuccino. Thereby, a nucleation phase is circumvented, leading to unhindered growth as expected^25–27^.

In contrast to Cappuccino, actin assembling on Spire-ABCD only formed smaller oligomers (Fig. 4E–H). This cannot be explained by photobleaching or other experimental settings. A simulation of a restricted growth process that stops after 4 monomers, considering the experimental determined labeling efficiency and photobleaching rate, could reasonably reproduce the key features of the measured data (Fig. 3E–H vs Fig. 4E–H). Therefore, Spire-ABCD can bind actin monomers but is insufficient to promote nucleation from monomers alone, as has been suggested before^33^. In the case of Spire-ABCD, the dwell-time analysis showed fast on-rates that increased with oligomer size, which would indicate a nucleation mechanism with fast polymerization (Fig. 4). Here, it was the combination of the visitation analysis and the average rate analysis that provided a meaningful interpretation of the data, which resembles a restricted growth process.

In summary, we developed new analysis methods for single and averaged filament data that can be obtained by fluorescence microscopy or other methods like iScat^12^. These analysis methods are highly robust towards low SNR and slow measurement rates, in contrast to the widely used dwell-time analysis. These methods will help to elucidate the processes involved in the very first stages of filament formation.

## Materials and methods

### Simulations

Monte Carlo simulations of protein assembly were performed with the given individual kinetic rates for each oligomer size. Starting from zero, single monomers were added or removed according to the association and dissociation rates for each step, thereby building up a monomer number versus time trace. The dwell-times for the addition or removal of monomers was drawn from an exponential distribution of dwell times corresponding to the inverse of the association or dissociation rates, respectively. Except for the first step, where no dissociation could occur, the decision of whether an association or dissociation event occurred next was given by the shorter of the randomly drawn dwell-time from the respective exponential distributions. The timing of the simulation then jumped directly to the next event according to a Gillespie algorithm. The monomer versus time trace was then built based on the monomer number and timing information. After simulating the monomer number versus time trace, Gaussian noise was added using different SNRs. An overview of the used simulation parameters (rates, sampling time, SNR, photobleaching rate and labeling efficiency) is given in Supplementary Table S1.

For simulating incomplete labeling of the monomers, the monomer number traces were modified by randomly determining the label number of labels for each step using a Poisson distribution of labeling efficiencies for stochastic labeling or a Bernoulli distribution for specific labeling. When incorporating photobleaching in the monomer versus time trace, down steps were introduced based on the simulated photobleaching rate. If a photobleached monomer dissociates, no down step occurs in the monomer number trace.

### Analysis

The noise-overlaid results of the simulations and the intensity measurements of the experimental data have been fed into a step-finding algorithm^34^. The position in time and the change in intensity associated with monomer addition or dissociation (steps) were identified by the Salapaka step-finding algorithm^34^. To prevent overfitting, the algorithm uses a penalty factor for the introduction of new steps^34^. The results of the step finding algorithm were converted into a monomer number versus time trace. Thereby, an up step was assumed to correspond to one binding monomer, a down step to one dissociating monomer.

For the experimental data, the individual intensity versus time traces were extracted from the intensity measured at the individual ZMW. Then, the intensity was converted into monomer numbers via a step-finding algorithm and an intensity to monomer conversion that also accounted for multiple steps via the step size distribution^6^.

### Dwell-time analysis

For comparison to our newly developed analysis tools, we investigated the distribution of dwell-times to calculate the stochastic rate constant from the fluorescence trajectory. For the dwell-time analysis at slow sampling rates, a double step correction was used. With slow sampling, fast individual steps cannot be resolved and appear as a single step with higher step sizes. For the double step correction, the mean step size per trace was calculated. An up or down step with a step size twice or three times the mean step size was treated as two or three monomers, respectively. The additional steps were assigned a dwell-time that corresponded to the interframe time of the measurement. This caused higher numbers in the first bin of the dwell-time histogram (Fig. S15). Therefore, the first bin was not included in the exponential fit.

### Experimental data

#### Labeled actin

Cy5-labeled actin (rabbit, skeletal muscle) was purchased from Hypermol (Bielefeld, Germany). We showed that fluorescently labeled actin is fully active as assayed by bulk techniques, TIRFM (Fig. S13) and single molecule methods^35^.

#### Expression and purification of Cappuccino and Spire constructs

Constructs encoding Spire-ABCD and Cappuccino, GST-ABCD1 and GST-CapuFH2^36^, were transformed in *E. coli* BL21-CodonPlus-RIL competent cells and cultured in LB medium with ampicillin (100 μg/mL) and chloramphenicol (34 μg/mL) at 37 °C. Protein expression was induced with 0.5 mM isopropyl-β-d-thiogalactopyranoside (IPTG) when OD600 reached a value of 0.6–0.8. Proteins were expressed for 16–20 h at 16 °C. Cells were harvested by centrifugation (30 min, 2000 rpm, 4 °C) and bacterial pellets were frozen at -20 °C.

Subsequently, pellets were handled on ice using ice-cold buffers. Pellets were thawed on ice and resuspended in ca. 50 mL of lysis buffer A (1×PBS, pH 7.4, 1 mM EDTA, 1 mM DTT, 0.5 mM PMSF) in the case of GST-ABCD1 or lysis buffer B (50 mM Tris–HCl pH 7.0, 150 mM NaCl, 0.2% Triton-X100, 1 mM DTT, 1 mM PMSF and 1 μg/mL DNAseI) in the case of GST-CapuFH2. After cell disruption by ultrasonification, the whole extract was clarified by centrifugation (30 min, 15,000 rpm, 4 °C). The soluble fraction containing recombinant proteins was then loaded onto a GSTPrep FF 16/10 column (GE Healthcare) connected to an ÄKTA FPLC System (GE Healthcare). After removal of bacterial host proteins, the column was washed with ten column volumes of PBS and then equilibrated with PreScission Protease buffer (50 mM Tris–HCl, pH 7.0, 150 mM NaCl, 1 mM EDTA, 1 mM DTT). Next, a solution of PreScission Protease (4 mg/mL) was loaded onto the column and incubated overnight at 4 °C. Subsequently, cleaved proteins were eluted from the column with PreScission Protease Buffer. The GST tag that remained on the column and PreScission Protease were removed by washing the column with regeneration buffer (50 mM Tris–HCl pH 8.0, 150 mM NaCl, 10 mM GSH). Fractions containing ABCD1 or CapuFH2 were concentrated and subsequently purified by a gel filtration method on a S75 Superdex column (ABCD1) or on a S200 Superdex column (CapuFH2) that was equilibrated with storage buffer (50 mM Tris–HCl, pH 8.0, 300 mM NaCl, 1 mM DTT).

#### Site-specific biotinylation via Sortase A-mediated ligation (SML)

Spire-ABCD and Cappuccino constructs were labelled at their N-termini using Sortase A-mediated ligation^36^. In a typical reaction, CapuFH2 (45 μM) or ABCD1 (50 μM) were mixed with an excess of desthiobiotin-peptide (300 μM) bearing a sequence recognized by Sortase A (desthiobiotin-GCGLPETGG, Smart Bioscience) and Sortase A (2 μM, Cat. #E4400-01; Eurx). The reaction mixture was supplemented with 10 mM CaCl_2_ and incubated for 6 or 24 h at 4 °C (in the case of ABCD1) or at 4 °C and 32 °C (for CapuFH2). The progress of the reaction was monitored with immunoblotting using HRP-conjugated streptavidin (Cat. #405210; BioLegend). After completion of the desthiobiotynilation reaction, both proteins were purified by size-exclusion chromatography. Desthiobiotin-CapuFH2 (DB-CapuFH2) was purified on a Superdex 200 Increase 10/300 column (GE Healthcare). The Desthiobiotin-ABCD1 construct was purified on a Superdex S75 column. The degree of desthiobiotinylation was calculated to be 53% and 60% for CapuFH2 and ABCD1, respectively as judged by an HABA assay (Pierce Biotin Quantification Kit, ThermoFisher Scientific).

#### Bulk assays

The biological activity of (DB-)CapuFH2 and (DB-)ABCD1, as well as functional actin growth were verified in bulk. Pyrene actin polymerization assays were done using 10% pyrene-labelled actin (C374) (PA, Hypermol) in black 96-well plates (Corning). Before the measurements, actin dissolved in G-buffer (Cytoskeleton, Inc.) was incubated in ME-buffer (50 µM MgCl_2_, 0.2 mM EGTA) for 5 min on ice. 80 µL of a 5.0 µM actin solution were placed in selected wells of the plate. Actin polymerization was induced by adding 20 µL of CapuFH2, DB-CapuFH2 or dmABCD1 in KMEI buffer (final concentrations: 5 mM KCl, 1 mM MgCl_2_, 0.5 mM EGTA and 0.2 mM imidazole). The final concentration of actin was 4 μM. The pyrene-fluorescence was excited at 350 nm and emission was measured at 520 nm on a TECAN microplate reader.

#### ZMW fabrication and functionalization

ZMWs were fabricated at the imec in Leuven, Belgium. In short, ZMWs were fabricated on glass coverslips (22mmx22mm, type #1, Menzel Glazer) by means of physical vapor deposition, e-beam lithography and low-pressure dry etching. ZMWs were passivated using 0.2% polyvinylphosphonic acid (Polysciences, USA) (10 min, 90 °C), followed by incubation in 3-[Methoxy(polyethyleneoxy)propyl]trimethoxysilane (6–9 PE-units, abcr, Karlsruhe, Germany) and PEG-biotin-silane (Nanocs Inc, New York, USA) in toluene (4 h, 55 °C). A schematic of the ZMW design can be found in Fig. S16.

#### Fluorescence-based microscopy and data extraction

Experiments were carried out on a home-built wide-field microscope system equipped with a 60 × 1.45 NA oil immersion objective (Plan Apo TIRF 60x; Nikon) (Fig. S17). The laser power of the 642 nm laser (06-01 MLD; Cobolt AB) was set to 2.8 mW before entering the objective via a dichroic mirror (zt405/488/561/640rpc; AHF Analysentechnik, Tübingen, Germany). The fluorescence signal was collected using the same objective, passed through an emission filter (680/42 BrightLineHC; AHF Analysentechnik, Tübingen, Germany) and recorded on an EMCCD camera (Andor iXon Ultra 888; Andor Technology) using an integration time of 50 ms.

The intensity as a function of time per aperture was extracted using a custom written software in MATLAB (The MathWorks) as described previously^6^. In short, the fluorescence signal of a signal mask matching the apertures was converted into an intensity versus time trace. After finding the up and down steps via a step-finding algorithm^34^, the intensity trace was converted into a monomer number trace (see^6^ for details), which was then used for further analysis.

#### Single-molecule imaging of actin polymerization in the presence of nucleators

Actin growth on Cappuccino and Spire-ABCD was measured in ZMWs. ZMWs were incubated with 0.15 mg/mL Streptavidin and 5 mg/mL BSA in PBS for 5 min. After washing with PBS, 30 nM biotinylated Cappuccino or Spire-ABCD was incubated for 5 min, followed by 5 min incubation with 1 mg/mL BSA and 1 mg/mL biotinylated BSA to block free streptavidin molecules on the surface. Actin was prepared as described in the following paragraph and added after washing with PBS.

Actin-Cy5 in G-buffer was incubated in magnesium exchange (ME) buffer for 5 min on ice (50 µM MgCl_2_, 0.2 mM EGTA, pH 7.0). Actin polymerization was induced by adding 1/10 volume of a 10 × concentrated KMEI buffer (final concentrations: 50 mM KCl, 1 mM MgCl_2_, 0.5 mM EGTA, and 0.2 mM imidazole at pH 7.0). The final buffer contained 800 nM actin and a PCA/PCD oxygen scavenging system with 250 nM protocatechuate dioxygenase ‘PCD’, 2.5 mM 3,4-dihydroxybenzoic acid ‘PCA’ and 1 mM Trolox^37^. After starting polymerization, 50 µL of the reaction mixture was added to the waveguides and data acquisition was started immediately with a measurement rate of 5 Hz, if not indicated otherwise. To measure the photobleaching rate, 1 µL of phalloidin was added to the waveguides after 1.5 h to stabilize the formed filaments and prevent dissociation.

#### TIRF imaging of actin filaments

TIRF microscopy of actin filaments was performed on the same setup as described above. The actin was treated in the same way as for the ZMW experiments, but instead of adding the solution onto the waveguides, a flow cell system was used as described in^31^. Thereby, an anchoring protein was attached to the glass surface of a cover slide. Thus, the attached actin filaments growing close to the glass surface can be imaged in TIRF mode. The length of the filaments was analyzed 250 s after induction of polymerization to verify the full functionality of actin-Cy5 by comparing the length distribution to actin-atto488 at the same time point after inducing polymerization (Fig. S13). Actin-atto488 was previously shown to be functional^6,35^.

## Supplementary Information

Supplementary Information.
