# Supplementary material for: Analysis tools for single-monomer measurements of self-assembly processes
Source: Sci Rep. 2022 Mar 18;12:4682. doi: 10.1038/s41598-022-08245-6 (PMC8933434; doi:10.1038/s41598-022-08245-6)
Supplement: Supplementary file 1 — Supplementary Information. [file 41598_2022_8245_MOESM1_ESM.docx]

Supplementary Material

Analysis tools for single-monomer measurements of self-assembly processes

Maria Hoyer^1^, Alvaro H. Crevenna^1,2^, Radoslaw Kitel^3,4^, Kherim Willems^5^, Miroslawa Czub^3^, Grzegorz Dubin^4^, Pol Van Dorpe^5^, Tad A. Holak^3^ and Don C. Lamb^1^*

^1^ Department of Chemistry, Center for NanoScience, Nanosystems Initiative Munich (NIM) and Center for Integrated Protein Science Munich (CiPSM), Ludwig-Maximilians University Munich, Munich, Germany.

^2^ Epigenetics and Neurobiology Unit, EMBL Rome, Monterotondo, Italy

^3^ Department of Organic Chemistry, Faculty of Chemistry, Jagiellonian University, Gronostajowa 2, 30-387 Krakow, Poland

^4^  Malopolska Centre of Biotechnology, Jagiellonian University, Gronostajowa 7a, 30-387 Krakow, Poland

^5^ imec, Kapeldreef 75, B-3001 Leuven, Belgium

* For correspondence: [d.lamb@lmu.de](mailto:d.lamb@lmu.de)

Supplementary Table S1: Table of simulation parameters for the simulations of a nucleation or conversion mechanism. The default settings are indicated in blue and the simulated range of the individual parameters is given in parenthesis. When a nucleation mechanism was simulated, all kinetics until the nucleus size is reached are governed by k^+^_nuc_ and k^-^_nuc_. After nucleation, the kinetics are given by k^+^_poly_ and k^-^_poly_. When a conversion mechanism was simulated, kinetics of association and dissociation rates from the conversion step size were given by k^+^_conv_ and k^-^_conv_. The kinetics of all other steps were given by k^+^_poly_ and k^-^_poly_ unless stated otherwise. When investigating the influence of the kinetic rates for both nucleation and conversion mechanisms, either only the association rates (change on-rate) or only the dissociation rates (change off-rate) were changed in comparison to the polymerization kinetics.

| k^+^_poly_ | 1 s^-1^ | |
| --- | --- | --- |
| k^-^_poly_ | **0.1 s^-1^** | |
| k_nuc_/k_conv_ | Change on-rate | Change off-rate |
| k^+^_nuc_/k^+^_conv_ | **0.5 s^-1^** (0.1 s^-1^ to 0.8 s^-1^) | **1** **s^-1^** |
| k^-^_nuc_/k^-^_conv_ | **0.1** **s^-1^** | **0.5 s^-1^** (0.2 s^-1^ to 1 s^-1^) |
| Nucleus/conversion size | **4** (2 to 5) | |
| SNR | **2** (0.1 to 3) | |
| Measurement rate | **20** s^-1^ (2 s^-1^ to 20 s^-1^) | |
| Photobleaching rate | **0** **s^-1^** (0.001 s^-1^ to 0.03 s^-1^) | |
| Labeling efficiency | specific: **1** (0.3 to 1), stochastic: 0.3 to 3 | |


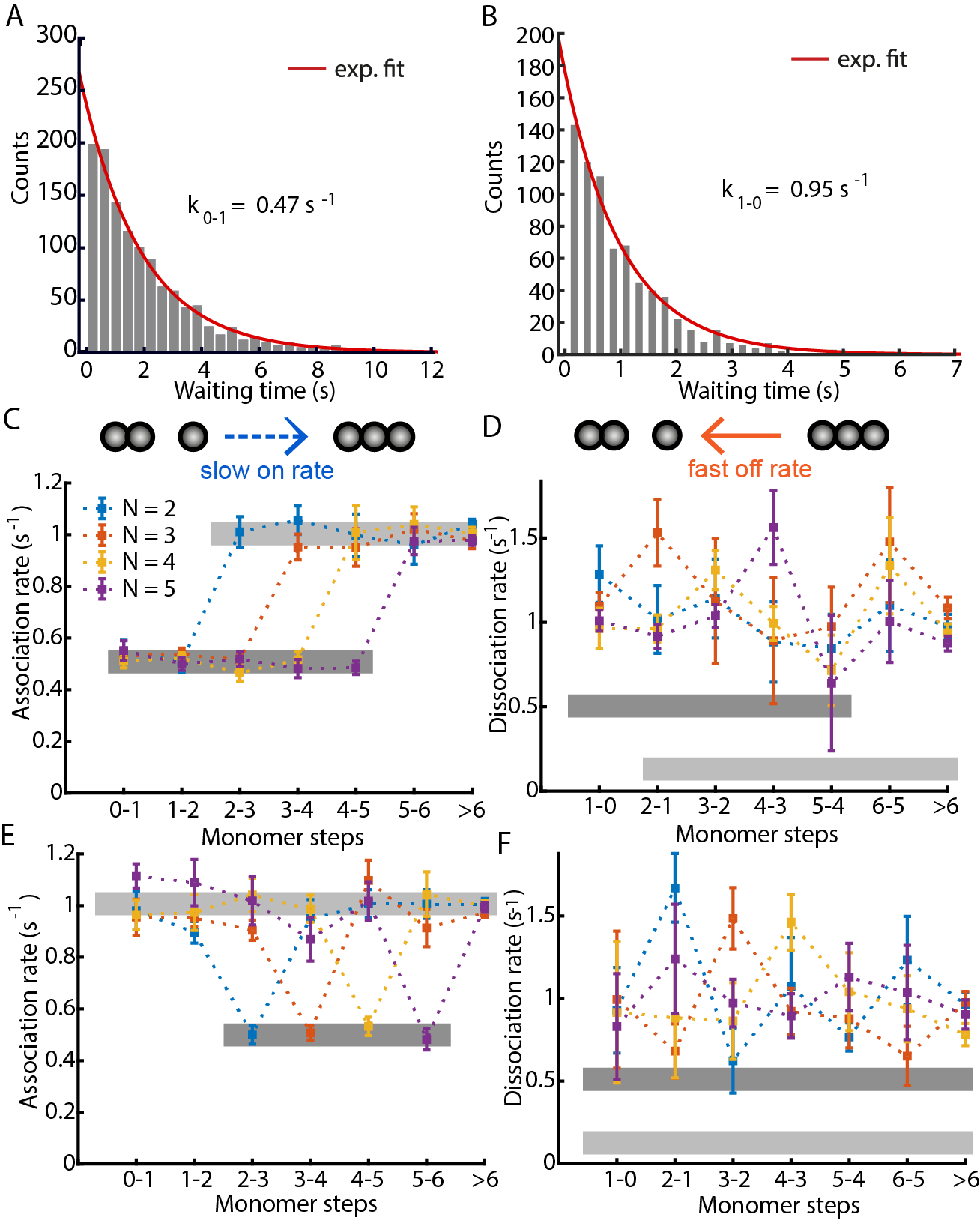


Figure S1: Dwell-time analysis on an assembly process with a conversion step at 2, 3, 4 or 5 monomers. (A, B): the dwell-time distributions for the first association step as simulated in (C) (A) and the first dissociation step as simulated in (D) (B) are shown. The dwell-time distribution for each individual step was fitted with a single-exponential distribution. (C, E): The extracted association rates for a nucleation (C) and a conversion mechanism (E) with different nucleation or conversion sizes. The dwell-time analysis is able to identify the association rates for the individual steps. The association rates during nucleation k^+^_nuc_ (indicated in dark gray) were set to 50% of k^+^_poly_ (indicated in light grey) with no change in the off-rates. (D, F): The extracted dissociation rates for a nucleation (D) and a conversion mechanism (F) with different nucleation or conversion sizes. The dwell-time analysis could not identify the dissociation rate changes during the nucleation or conversion steps. The dissociation rates during nucleation k^-^_nuc_ (indicated in dark grey) were chosen to be 5x of k^-^_poly_ (indicated in light grey) with no change in the on-rates. Error bars for the rates represent the 95% confidence intervals of the exponential fits.


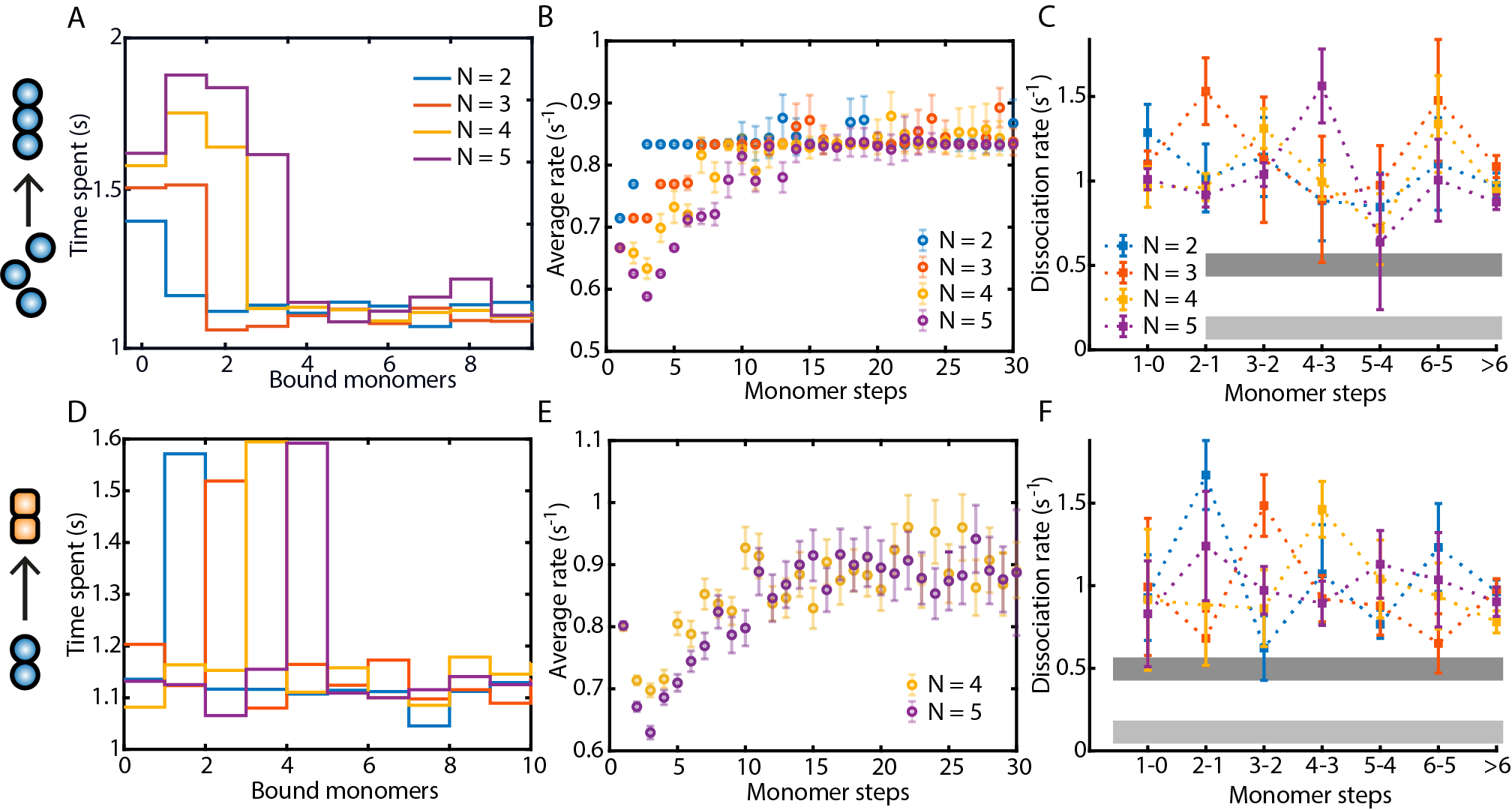


Figure S2: A comparison of the visitation analysis, average rates and dwell-time analysis on an assembly process with nucleus or conversion size of 2, 3, 4 or 5 monomers. The dissociation rates during nucleation k^-^_nuc_ (indicated in dark grey in C and F) were chosen to be 5x faster than k^-^_poly_ (indicated in light grey in C and F) with no change in the on-rates. (A, D): A visitation analysis on a nucleation (A) and on a conversion mechanism (D). (B, E): Average rate analysis on the same nucleation and conversion mechanism as in (A, B). (C, F): A dwell-time analysis on the same nucleation and conversion mechanism as in (A, B). The dwell-time analysis cannot identify a mechanism with a change in the off-rate, since the extracted dissociation rates do not indicate any changes in the simulated rates. Error bars for the rates represent the 95% confidence intervals of the exponential fits.


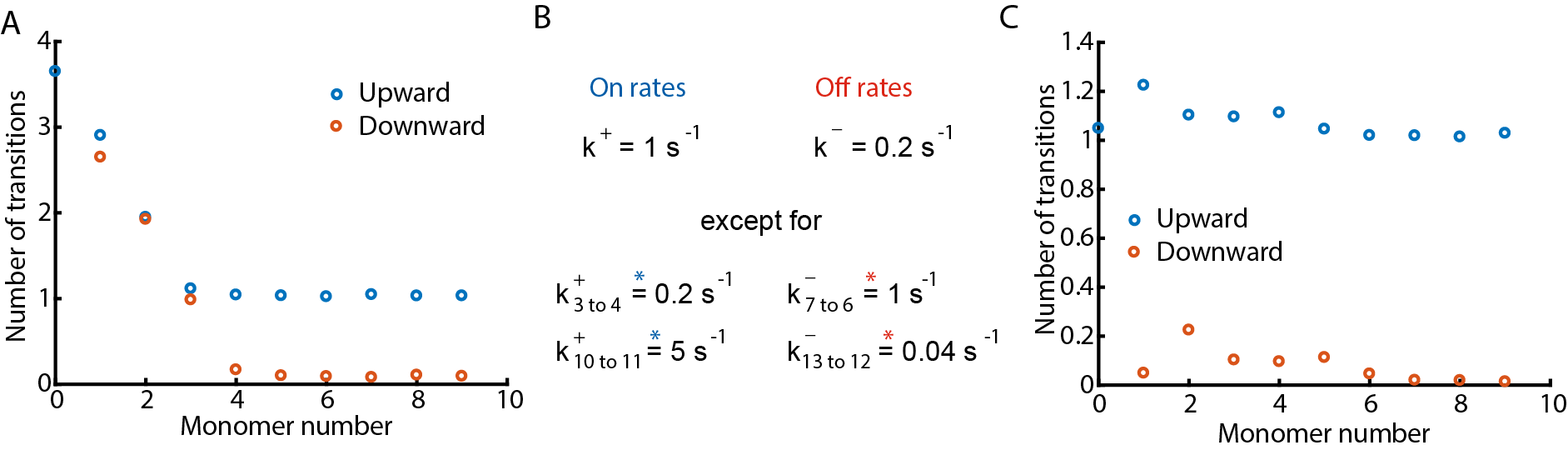


Figure S3: Mean number of upward (blue) and downward (red) transitions from a certain oligomer size (monomer number). A: Calculation for a nucleation mechanism with a nucleus size of 3. During nucleation, many upward and downward transitions occur. When the nucleus size is overcome, i.e. during polymerization, mainly upward transitions take place. B: A model for a hypothetic growth mechanism with different rates as shown. C: Calculation of the mean number of upward (blue) and downward (red) transitions from a certain oligomer size (monomer number) using the kinetic model shown in panel B. The number of upward and downward transitions is not sensitive enough to detect kinetic changes in single steps.


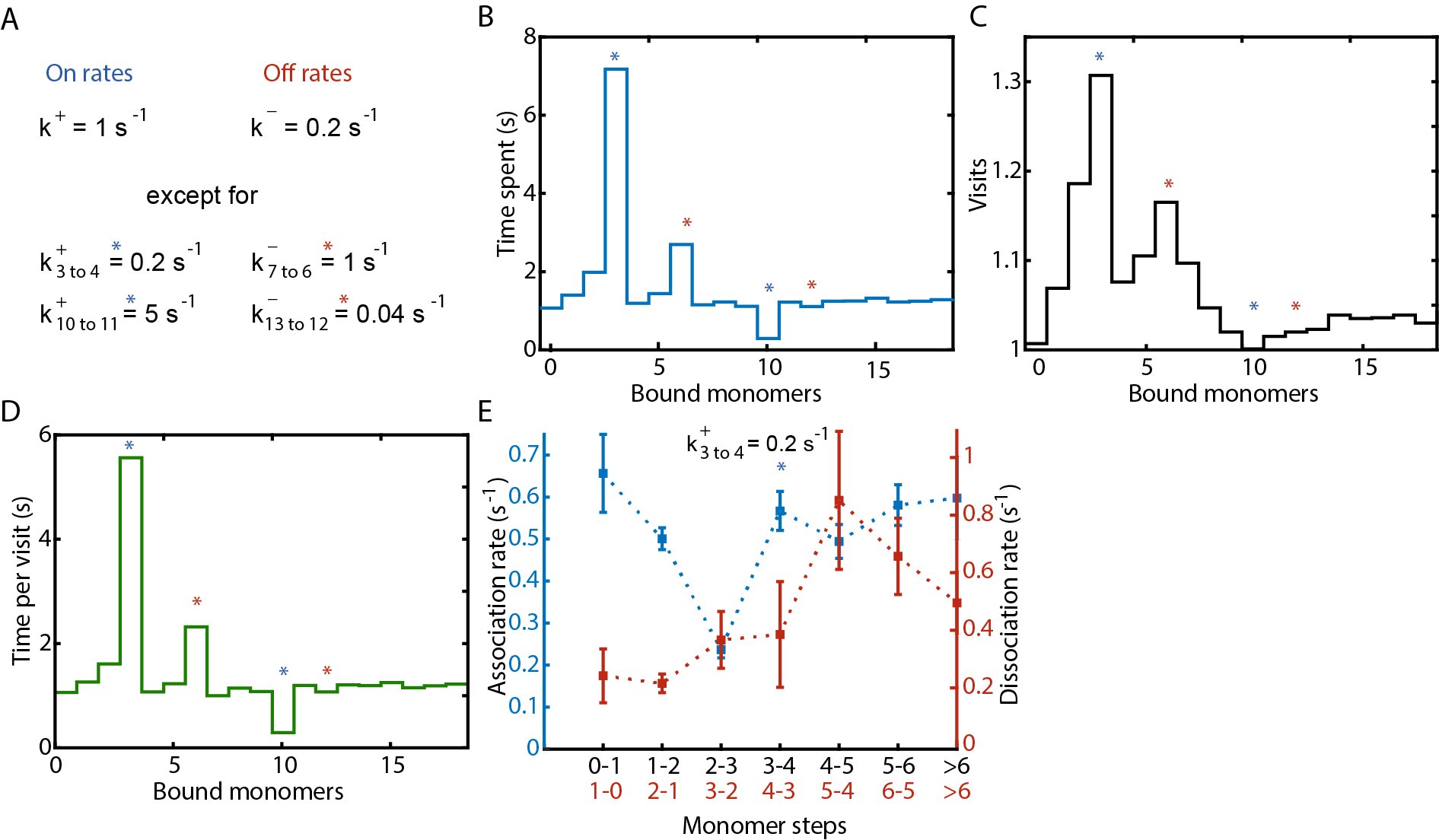


Figure S4: A visitation analysis on a growth process where single steps have different kinetics. A: Model used for the simulation. All monomer binding events have an on-rate k^+^ = 1 s^-1^, all dissociation events an off-rate k^-^ = 0.2 s^-1^ with the following exceptions: The third and 10^th^ monomer binding event show a slower or a faster on-rate, respectively (by a factor of five, if not otherwise indicated) and are marked by blue stars; the 7^th^ and 13^th^ monomer binding event show a faster or slower off-rate, respectively, and are marked by red stars. All rates are simulated to show a single-exponential distribution. B-D: The visitation analysis can resolve multiple single steps with different kinetics. B: The mean time the traces spent at a certain oligomer size is plotted. C: The mean number of occurrences is plotted as a function of oligomer size (i.e., number of visits). D: The mean time per visit is plotted as a function of oligomer size. E: Dwell-time analysis on the same simulated growth process. The dwell-time analysis is not able to resolve multiple individual rates even with a SNR of 2, measurement rate of 20 s^-1^ and no photobleaching. For the dissociation rates, the dwell-time analysis was shown to be unreliable for determination of the growth mechanism where the association rates are always faster than the dissociation rates (Figure S1). For multiple steps with different kinetics as simulated here, also the association rates cannot be extracted reliably.


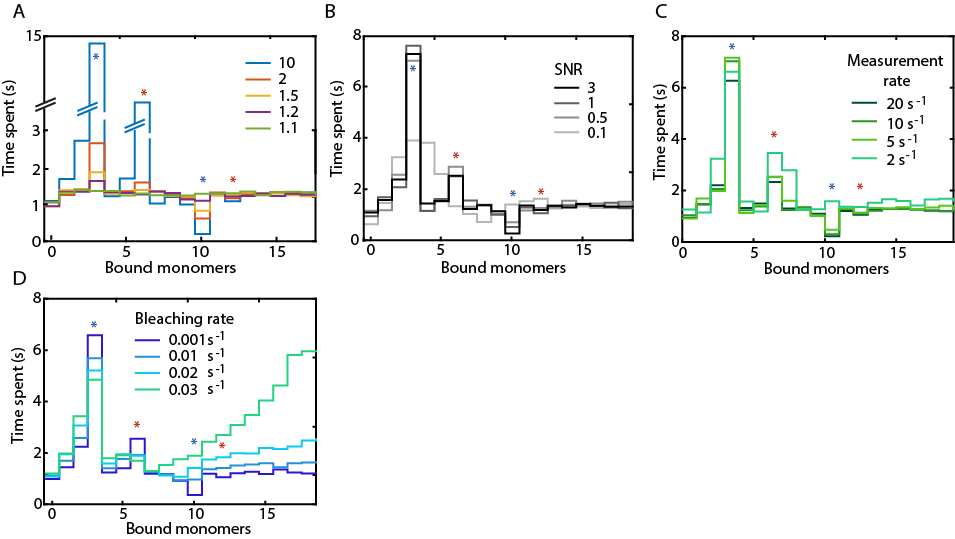


Figure S5: Reliability of the visitation analysis for a hypothetic growth process. (A): A visitation analysis of simulations using the model described in Figure S4A, where the varying the default rates by different factors. The difference between the single steps with slower or faster kinetics can still be detected for differences of a factor of 1.5. (B): Impact of the SNR. Using a difference in kinetic rates of a factor of five, we simulated SNR ratios from 3 to 0.1. Individual steps are visible down to a SNR of 0.5. (C): Varying the sampling rate. Using the model described in Figure S4A, we performed simulations with different sampling times. The visitation analysis is able to detect individual steps at measurement rates down to 5 times the default on-rate. (D): Visitation analysis on simulations considering the influence of photobleaching. The association rates during nucleation need to be fast compared to the photobleaching rate in order to resolve the first steps in an assembly process.


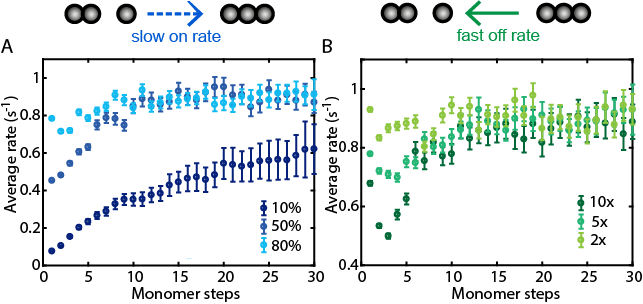


Figure S6: The influence of a change in the on-rate versus a change in the off-rate in the average rate analysis for a nucleation mechanism with a nucleus size of 4 monomers. (A): Average rate analysis on simulations varying the association rate during nucleation. The association rates during nucleation k^+^_nuc_ were set to 10, 50 or 80% of k^+^_poly_. (B): Average rate analysis on simulations varying the dissociation rate during nucleation. The dissociation rates during nucleation k^-^_nuc_ were a factor of 10, 5 or 2 of k^-^_poly_. The average rates show a different behavior dependent on whether the association rate or the dissociation rate was changed with respect to the polymerization rates.


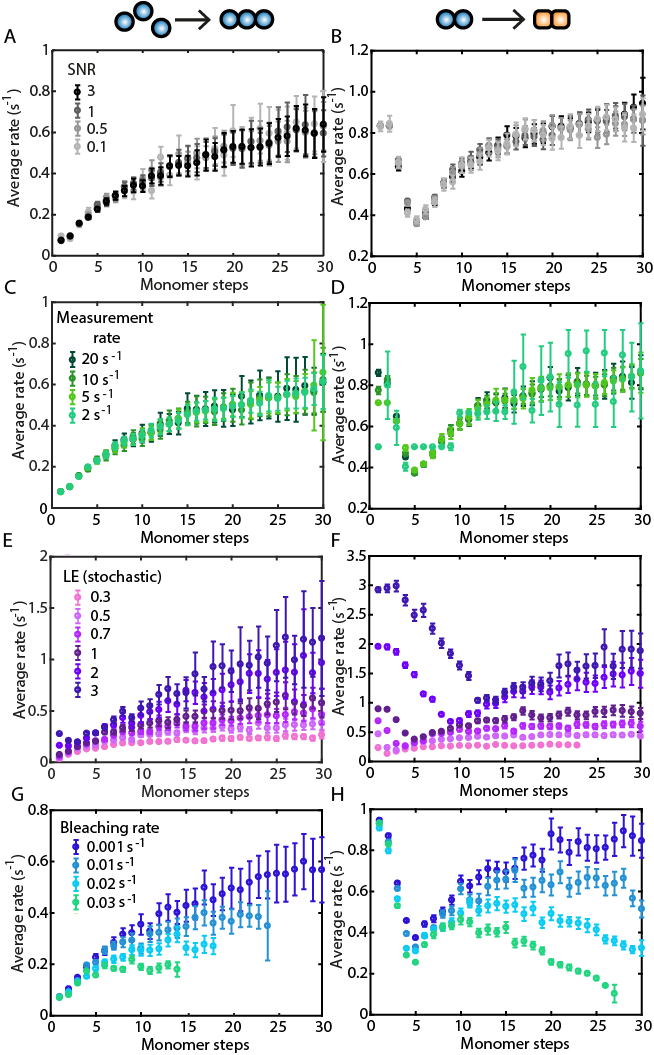


Figure S7: Robustness of the average rate analysis. (A, B): An average rate analysis on simulations applying different SNRs. A nucleation mechanism (A) or a slow conversion step (B) can be detected even at a SNR of 0.1. (C, D): An average rate analysis on simulations varying the sampling time. To detect a nucleation mechanism or conversion step, the measurement rates should be at least 2x faster than the fastest rate. (E, F): An average rate analysis for simulations using monomers with different labeling efficiencies. A nucleation mechanism (E) or a conversion step (F) can be detected at all tested labeling efficiencies down to 30%. For the correct conversion step (F), the labeling efficiency should be at 100%. (G, H): An average rate analysis for simulations considering the influence of photobleaching. A high photobleaching rate can shorten the average trace or lead to apparently slower kinetics.


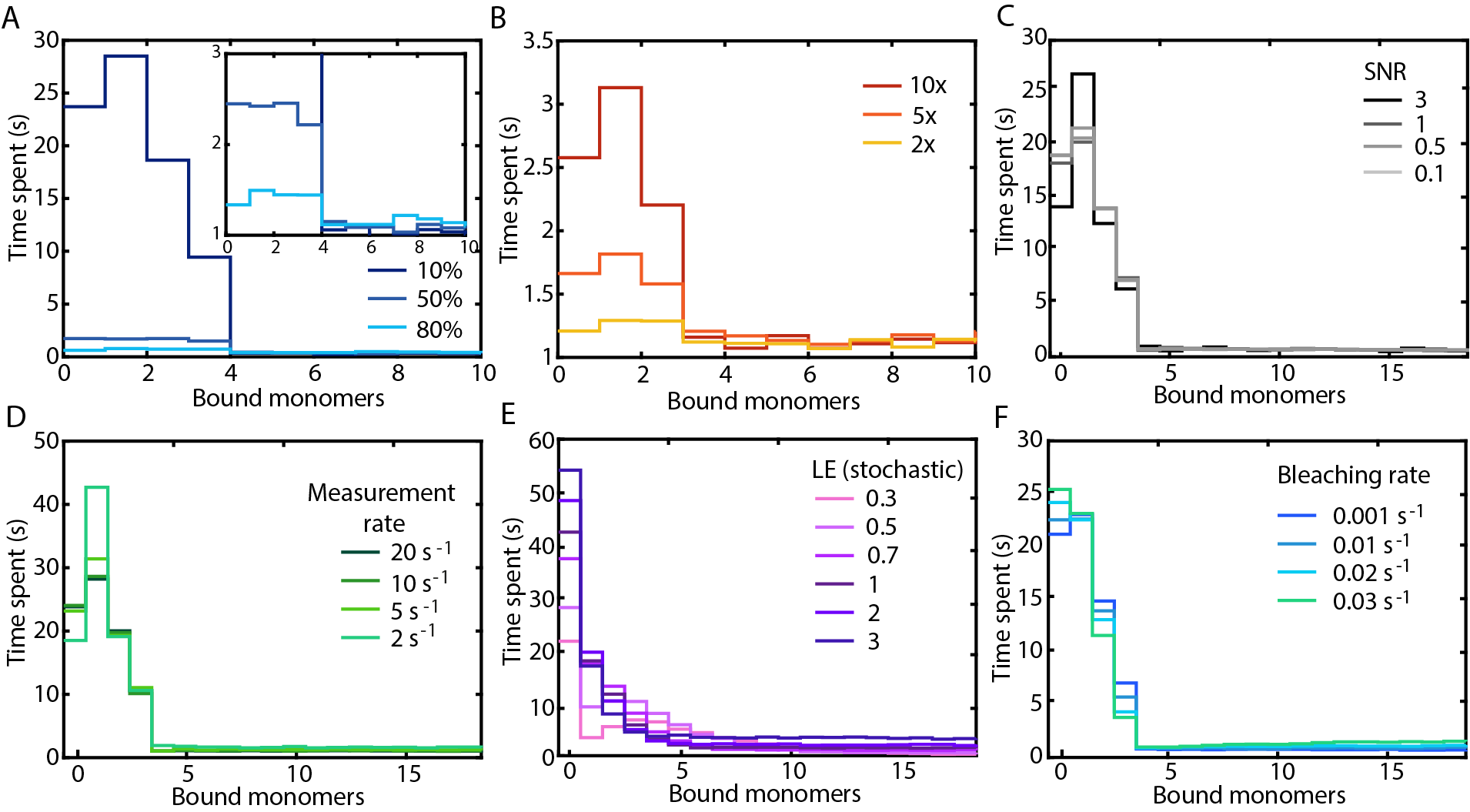


Figure S8: Robustness of the visitation analysis applied to a nucleation mechanism with a nucleus size of four monomers. (A, B): Difference between nucleation and polymerization kinetics. The association rates during nucleation k^+^_nuc_ were chosen to be 10, 50 or 80% of k^+^_poly_ (A). The dissociation rates during nucleation k^-^_nuc_ were a factor of 10, 5 or 2 of k^-^_poly_ (B). (C): Visitation analysis on simulations varying the SNR. A nucleation mechanism can be detected until a SNR of 0.1. (D): Visitation analysis on simulations varying the sampling time. The visitation analysis is quite robust towards slow measurement rates. (E): Visitation analysis on simulations using monomers with different degrees of stochastic labeling. The stochastic labeling efficiency (LE) should be slightly below 100% for the correct estimation of the nucleus size. (F): Visitation analysis of simulations considering the influence of photobleaching. The early steps during nucleation can be resolved even at a photobleaching rate of 3% of the association rate.


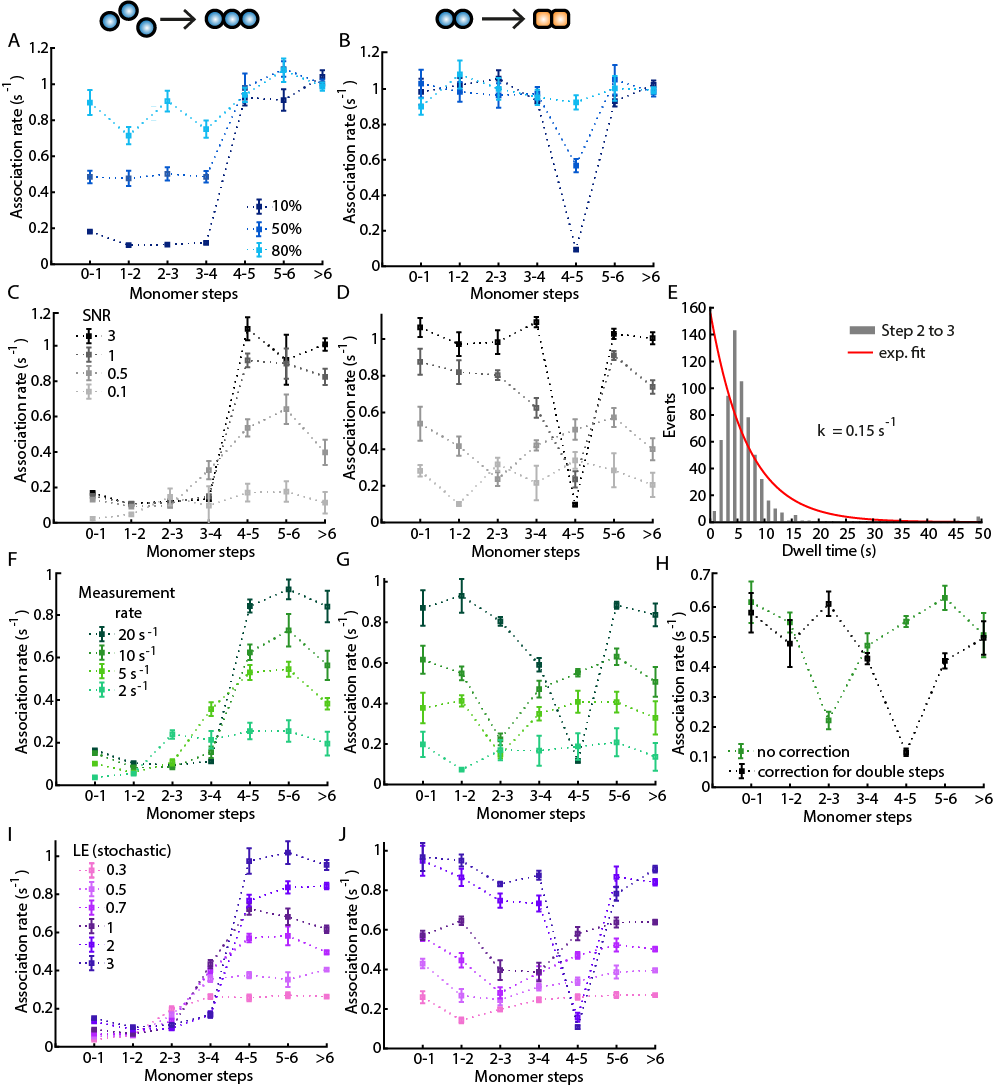


Figure S9: Robustness of the dwell-time analysis. For all simulations, a nucleus or conversion step at four mononomers was chosen. (A, B): Extracted on-rates for simulations varying the difference between nucleation and polymerization kinetics. The association rates k^+^_nuc_ and k^+^_conv_ were chosen to be 10, 50 or 80% of k^+^_poly_. (C, D): Extracted on-rates for simulations applying different SNRs. A nucleation mechanism (C) or a slow conversion step (D) can be detected until a SNR of 1. (E): With low SNR, fast steps are not detected and the dwell-time distribution does not follow an exponential decay (red line). (F, G): Extracted on-rates for simulations varying the sampling time. To estimate association rates, the measurement rates should be at least 10x faster than the fastest rate. (H): By using a correction for fast subsequent steps that are detected as single steps with larger amplitude, the correct conversion step can be determined despite a measurement rate of 10 s^-1^ or kinetics rates 10x slower than the sampling rate. (I, J): Extracted on-rates for simulations of monomers with different degrees of labeling. A high labeling efficiency (LE) is needed for correct interpretation of the dwell-time analysis. For stochastic labeling, the LE should ideally be more than 100%.


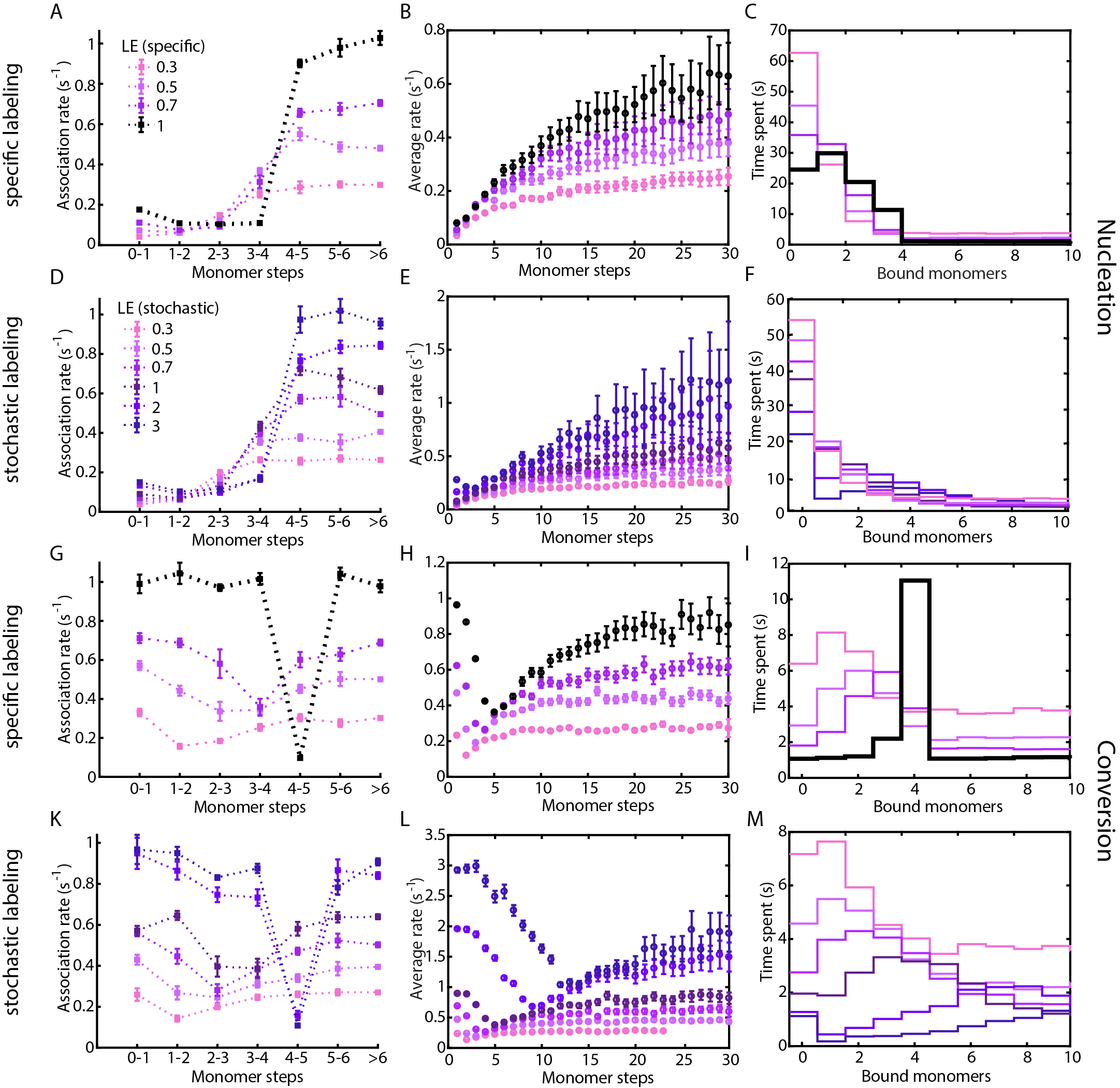


Figure S10: A comparison of the effect of stochastic and specific labeling efficiencies on the different analysis methods. (A-F): A dwell time analysis (A, D), average-rate analysis (B, E) and a visitation analysis (C, F) for simulations of a nucleation mechanism assuming incomplete specific labeling (A-C) or different degrees of stochastic labeling (D-F). (G-M): A dwell time analysis (G, K), average-rate analysis (H, L) and a visitation analysis (I, M) for simulations of a conversion mechanism assuming incomplete specific labeling (G-I) or different degrees of stochastic labeling (K-M). For specific labeling, there is either 1 or 0 labels per monomer while, for stochastic labeling, multiple labels per monomer are possible. The ideal case, i.e. specific labeling with 100% labeling efficiency, is indicated in black (A-C and G-I). For a nucleation mechanism, a wide range of labeling efficiencies can be tolerated for all analysis methods. However, the dwell-time analysis extracts the correct polymerization rates only for labeling efficiencies of 100% for specific labeling (A) or more than 100% for stochastic labeling (D). For specific labeling with a labeling efficiency of less than 100%, the polymerization rate can be estimated since it is given by the extracted polymerization rate multiplied by the labeling efficiency; LE*k_poly_ (panel A)”. For the determination of the correct conversion size, the labeling efficiency should be at 100% for the average rate analysis and the visitation analysis. For the dwell-time analysis, labeling efficiencies at or above 100% are needed.


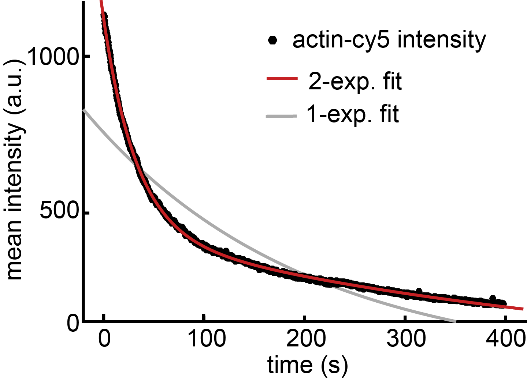


Figure S11: Decay of the average intensity of actin-Cy5 stabilized with phalloidin (see materials and methods for details). A single exponential fit (grey) does not describe the data, but a double-exponential decay (red) does. The decay constants are 0.025 s^-1^ and 0.0015 s^-1^.


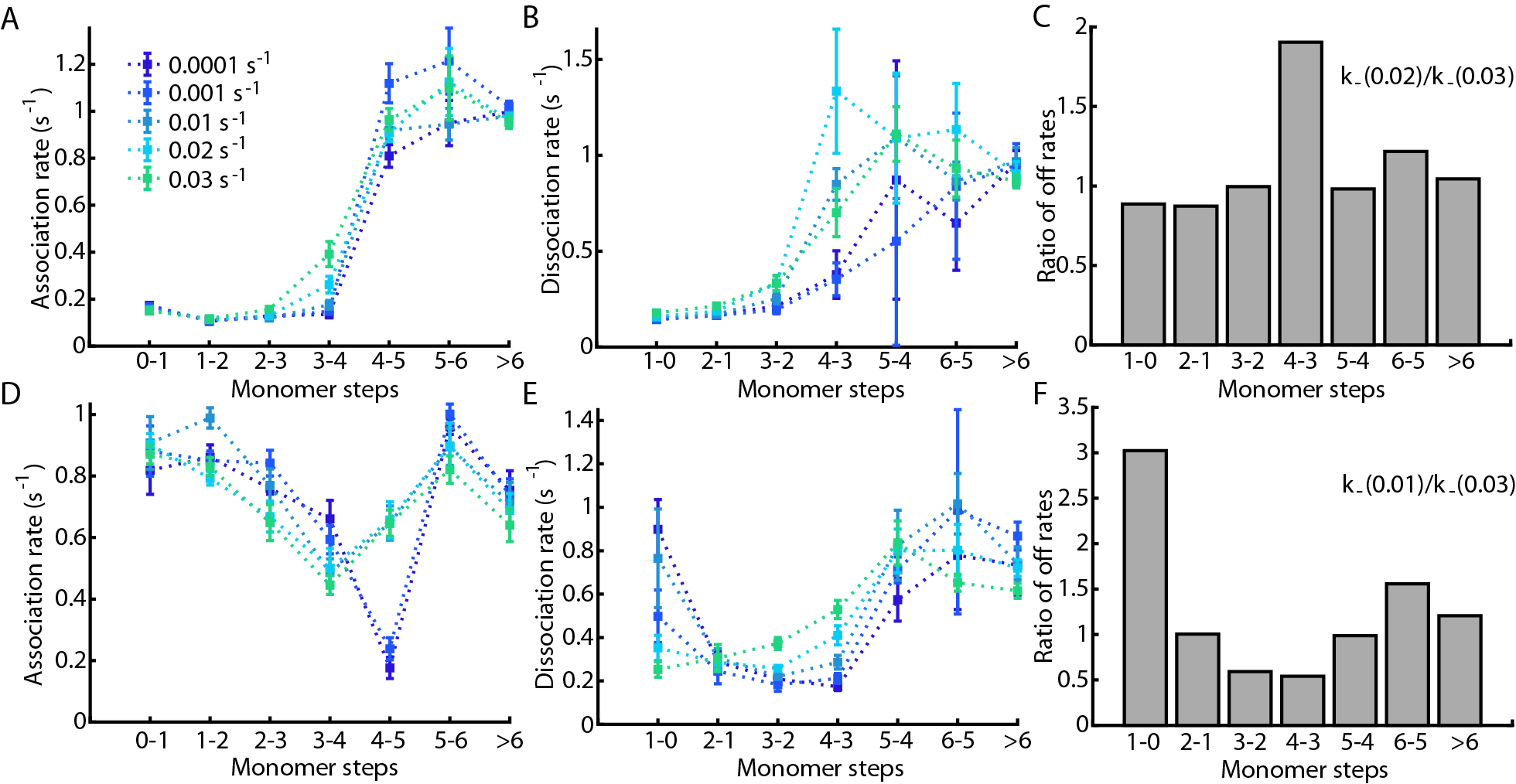


Figure S12: Using photobleaching as a tool to estimate the nucleation or conversion step. Dwell-time analysis on a simulation assuming a nucleation mechanism (A-C) or a conversion mechanism (D-F) with k^+^_nuc_ = 0.1 k^+^_poly_ and no change in the off-rates. Photobleaching with different photobleaching rates has been applied to the simulations. (A, B): The extracted association rates are plotted and the nucleation or conversion step at 4 monomers is observable. (B, E): The extracted dissociation rates are plotted for the same simulated processes as in (A) and (D). The apparent dissociation rates at the nucleus or conversion size are affected most by different photobleaching rates. (C, F): The ratio between the apparent dissociation rates for the same simulation under the influence of different photobleaching rates are plotted for each step. The difference between the apparent dissociation rates is the highest at the nucleation or conversion size.


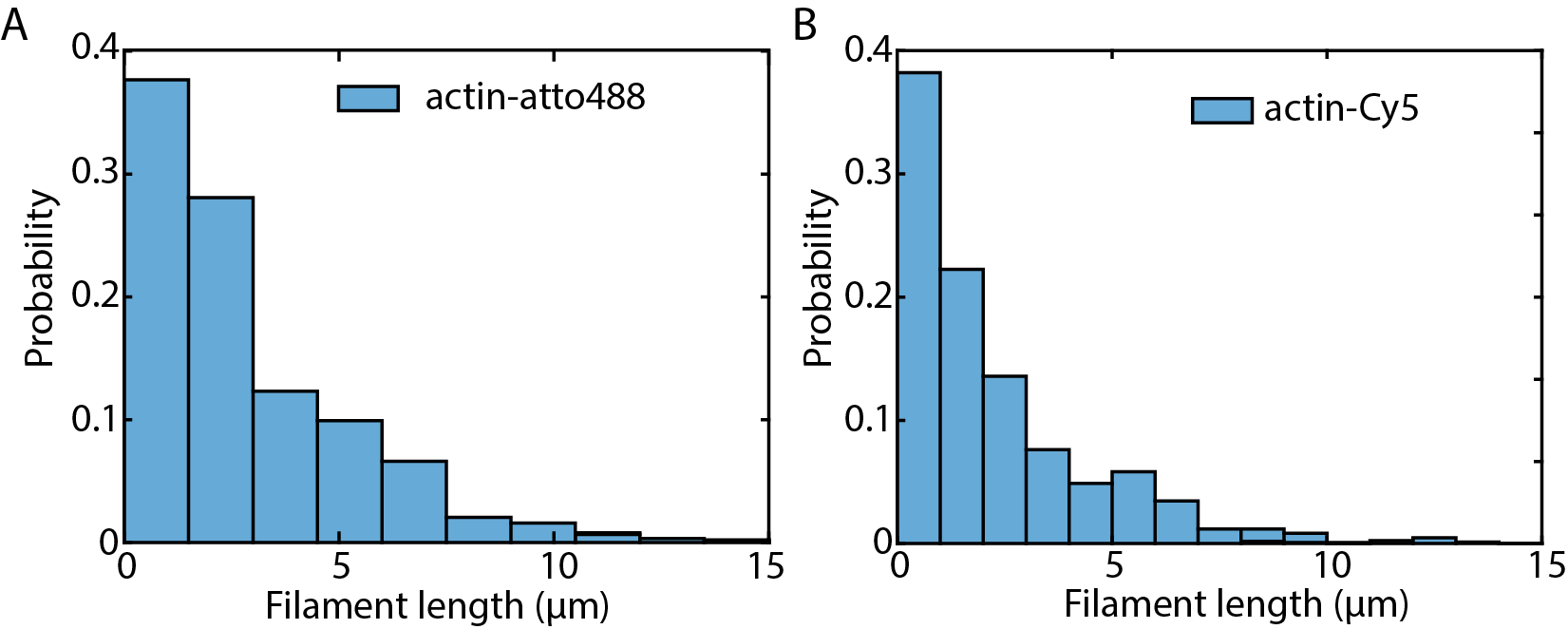


Figure S13: Functionality of Actin-Cy5. Using TIRF microscopy, we tested the functionality of labeled Actin by measuring the filament length distribution (see materials and methods for details). A: Filament length distribution 250 s after induction of polymerization of 30% labeled actin-atto488. We have previously shown that Actin-atto488 is functional (*6, 35*). B: The filament length distribution of actin-Cy5 250 s after induction of polymerization. Actin-Cy5 shows a similar length distribution as actin-atto488, indicating that labeling with Cy5 also does not impair the functionality of actin.


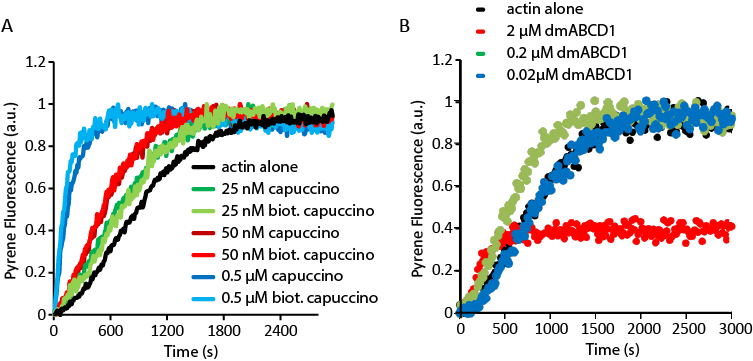


Figure S14: Biotinylated Cappuccino and dmABCD1 is functional as monitored by a pyrene assay. A: The nucleation rate of actin alone (black) is enhanced by Cappuccino (dark green, red and blue) and biotinylated Cappuccino (light green, red and blue) in a concentration-dependent manner. B: The nucleation rate of actin alone (black) is enhanced by 0.2 (green) and 2 µM (red) dmABCD1.


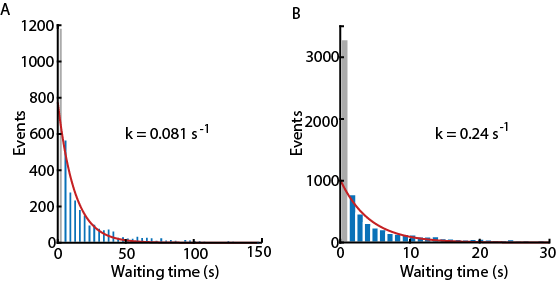


Figure S15: Waiting time distribution of Cappuccino-mediated growth with exponential fit (red) of all steps measured with (A) 5 Hz or (B) 16.7 Hz. The first bin (grey) was not included in the fit, since it shows higher numbers because of the double step correction, where unresolved steps get the interframe time of the measurement as dwell time.


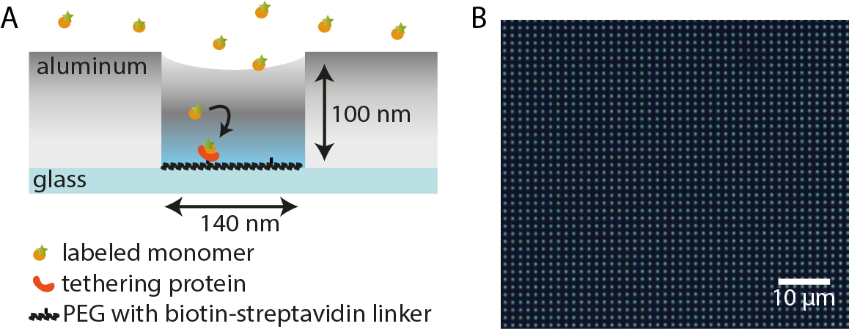


Figure S16: ZMWs used for measuring protein assembly and bright field image of ZMWs. (A) Schematic of an aperture from the ZMW. An aluminum layer deposited on a cover glass is etched to form circular apertures. Inside the apertures, an evanescent field is created when the structure is illuminated from below. The bottom of the ZMWs, i.e. the glass surface, is functionalized with PEG and biotinylated PEG, which allows binding of a biotinylated tethering protein (orange) via a biotin-streptavidin-biotin linkage. The aluminum surface is passivated as described in the materials and methods section. Fluorescently labeled monomers (yellow) bind to the tethering protein and the assembly process begins. (B) A bright field image of an array of ZMWs.


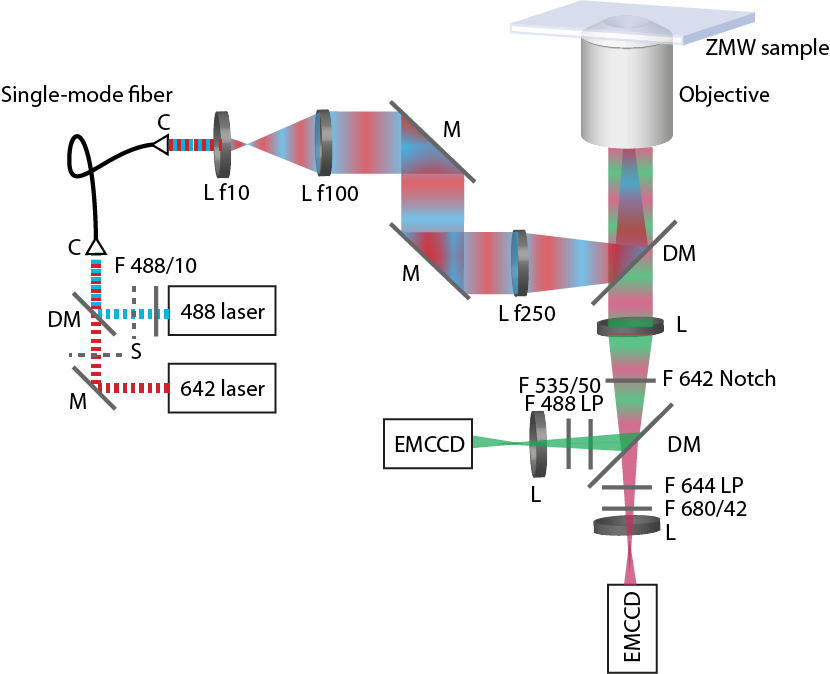


Figure S17: Schematic of the experimental setup. For excitation, a 488 nm laser (06-01-MDL, Cobolt AB) and a 642 nm laser (06-01-MDL, Cobolt AB) are coupled into a single-mode fiber (SM450, Thorlabs) and controlled individually via shutters (SHB025T, Thorlabs). After the fiber, the beam size was expanded 10-fold by a telescope (L f10 and L f100). By fixing a mirror and a tube lens on a movable stage, the setup could be used both in TIRF mode and in wide-field mode for ZMW illumination. The light is guided into the objective (60x 1.45 NA oil immersion objective Plan Apo TIRF 60x, Nikon) via a dichroic mirror (zt405/488/561/640rpc, F73-410, AHF Analysentechnik) and onto the ZMW. The fluorescence is collected by the same objective and transferred through a 642 Notch filter (642 Notch, AHF Analysentechnik) onto a dichroic mirror (FF580-FDi01, Andor Technology) to separate green and red fluorescence. The green fluorescence emission passed through two fluorescence emission filters (535/50 and 488 LP, AHF Analysentechnik) because of the high reflection intensity of the laser light caused by the aluminum surface of the ZMW. The red fluorescence was passed through a 680/42 emission filter (AHF Analysentechnik) and a 644 LP long pass filter (AHF Analysentechnik). For the data presented here, only the blue pathway was used. The signal was recorded by two EMCCD cameras (Andor iXon Ultra 888, Andor Technology) with 1024x1024 pixels per chip. Abbreviations: M: mirror, DM: dichroic mirror, C: collimator, L: lens, F: filter, S: shutter.
